# A Simple RNA Target Capture NGS Strategy for Fusion Genes Assessment in the Diagnostics of Pediatric B-cell Acute Lymphoblastic Leukemia

**DOI:** 10.1097/HS9.0000000000000250

**Published:** 2019-05-22

**Authors:** Andrea Grioni, Grazia Fazio, Silvia Rigamonti, Vojtech Bystry, Giulia Daniele, Zuzana Dostalova, Manuel Quadri, Claudia Saitta, Daniela Silvestri, Simona Songia, Clelia T. Storlazzi, Andrea Biondi, Nikos Darzentas, Giovanni Cazzaniga

**Affiliations:** 1 Centro Ricerca Tettamanti, Clinica Pediatrica, Università degli Studi di Milano-Bicocca, Fondazione MBBM/Ospedale S. Gerardo, Monza, Italy; 2 Central European Institute of Technology, Masaryk University, Brno, Czech Republic; 3 Department of Biology, University of Bari “Aldo Moro”, Bari, Italy; 4 Department of Hematology, University Hospital Schleswig-Holstein, Kiel, Germany; 5 Clinica Pediatrica, Università degli Studi di Milano-Bicocca, Fondazione MBBM/Ospedale S. Gerardo, Monza, Italy; 6 Cancer Center, Humanitas Research Hospital, Humanitas University, Rozzano, Milan, Italy; 7 Center of Biostatistics for Clinical Epidemiology, Department of Health Science, University of Milano-Bicocca, Milan, Italy; 8 Pediatric Hematology-Oncology Unit, Department of Pediatrics, University of Milano-Bicocca, MBBM Foundation/ASST Monza, Monza, Italy.

## Abstract

Acute lymphoblastic leukemia (ALL) is the most frequent pediatric cancer. Fusion genes are hallmarks of ALL, and they are used as biomarkers for risk stratification as well as targets for precision medicine. Hence, clinical diagnostics pursues broad and comprehensive strategies for accurate discovery of fusion genes. Currently, the gold standard methodologies for fusion gene detection are fluorescence in situ hybridization and polymerase chain reaction; these, however, lack sensitivity for the identification of new fusion genes and breakpoints. In this study, we implemented a simple operating procedure (OP) for detecting fusion genes. The OP employs RNA CaptureSeq, a versatile and effortless next-generation sequencing assay, and an in-house as well as a purpose-built bioinformatics pipeline for the subsequent data analysis. The OP was evaluated on a cohort of 89 B-cell precursor ALL (BCP-ALL) pediatric samples annotated as negative for fusion genes by the standard techniques. The OP confirmed 51 samples as negative for fusion genes, and, more importantly, it identified known (*KMT2A* rearrangements) as well as new fusion events (*JAK2* rearrangements) in the remaining 38 investigated samples, of which 16 fusion genes had prognostic significance. Herein, we describe the OP and its deployment into routine ALL diagnostics, which will allow substantial improvements in both patient risk stratification and precision medicine.

## Abstract 2

Supplemental Digital Content is available in the text

## Introduction

Acute lymphoblastic leukemia (ALL) is the most common pediatric cancer.^1^ The 5-year survival rate exceeds 85% in children, but the survival following relapse is poor.^2^ Analysis of paired diagnosis/relapse ALL samples shows clonal diversity that arises from the accumulation of new deletions and mutations over time. Despite that, the founding fusion genes are usually conserved from diagnosis to relapse, indicating that the predominant clones observed at diagnosis and relapse are clones derived from a common ‘preleukemic’ clone.^3^ Fusion genes arise from chromosomal translocations and intrachromosomal rearrangements that mainly disrupt genetic regulators of normal hematopoiesis as well as lymphoid development (e.g., those involving *RUNX1* and *ETV6*) and constitutively activate tyrosine kinases^4^ (e.g., *ABL1* chimeras). Thus, fusion genes are hallmarks of ALL that play a pivotal role in leukemogenesis, and their identification is crucial for patient risk stratification.^5^

Common fusion genes in B-lineage ALL are: t(12;21)(p13;q22), encoding ETV6-RUNX1 (TEL-AML); t(1;19)(q23;p13), encoding TCF3-PBX1 (E2A-PBX1)^6^; t(9;22)(q34;q11.2), resulting in formation of the “Philadelphia” chromosome, encoding BCR-ABL1; rearrangements of *KMT2A* (*MLL*) at 11q23 to a range of fusion partners^7^; and rearrangements of the cytokine receptor gene *CRLF2* at the pseudo autosomal region 1 (PAR1) at Xp22.3/Yp11.3.^8,9^ Fusion genes correlate with the clinical outcome, and they are used as biomarkers for patient risk stratification^10^: for example, patients positive for t(12;21)/ETV6-RUNX1 have the most favorable prognosis, whereas t(9;22)/BCR-ABL1, t(1;19)/TCF3-PBX1, and KMT2A-AFF1 correlate with a brief disease latency and have a poor prognosis.^10,11^ Moreover, specific drug inhibitors antagonizing the fusion proteins provide a more efficient and less toxic tool for disease eradication (precision medicine): for example, the imatinib tyrosine kinase inhibitor inhibits the oncogenic deregulation caused by the (9;22)/BCR-ABL1 fusion protein.^12^

Before the next generation sequencing (NGS) era, elaborate and extensive cytogenetic studies lead to the description of few recurrent and highly expressed fusion genes,^13^ such as BCR-ABL1 and ETV6-RUNX1. The characterization of their breakpoint coordinates enabled the design of diagnostic screening by both quantitative multiplex polymerase chain reaction (qPCR) and fluorescence in situ hybridization (FISH).^14^ The recent introduction of NGS allowed a fast and accurate screening of the patient's genome at the nucleotide level, which lead to the discovery of a broad array of previously unknown fusion genes.^15^ This reflects the increased capability of NGS to recognize subtle chromosomal rearrangements. On the contrary, FISH may only detect exchanges of considerably larger chromosome segments, without nucleotide precision, while qPCR screenings can identify already known fusion gene breakpoints only.^16^

Whole transcriptome sequencing (RNAseq), together with open-source bioinformatics tools, has already been applied to identifying fusion genes.^17^ Whole RNAseq performs well in the detection and quantification of highly and medium abundant transcripts, but it may fail in cases of low abundance transcripts.^18^ The RNA capture sequencing (RNA CaptureSeq) is a probe-based assay for capturing, amplifying, and sequencing genomic regions of interest only (targets). The RNA CaptureSeq generates libraries of small fragments (250–300 bp) in a short time (2.5 days) compared to whole RNAseq, and it is compatible with the well-known MiSeq and NextSeq Illumina NGS platforms. RNA CaptureSeq is sensitive to low abundance transcript variants of targeted genes^19^; however, the detection of fusion transcripts may be compromised when the fusion partner gene is not part of the capture procedure (unknown partner). This scenario reduces discoverability of fusion transcripts to only those fragments that span the target gene breakpoint.

We have developed and herein present a simple, efficient, and ready-to-use operating procedure (OP) for the clinical identification of fusion genes in B-cell ALL. The OP is based on RNA CaptureSeq, and it is supported by an in-house bioinformatics pipeline that is purpose-built to detect and extend fragments spanning the fusion gene breakpoint. We applied the OP to a cohort of 89 B-cell ALL pediatric patients enrolled in the AIEOP-BFM ALL clinical protocol^20^ that were annotated as negative to fusion genes by the standard screening methods. This paper summarizes the results of the OP applied to clinical diagnostics and discusses its implications for patient risk stratification.

## Results

### Comparison of available bioinformatics pipelines

We developed a bioinformatic method for fusion gene assessment from RNA CaptureSeq datasets and evaluated it on a training dataset composed of 23 samples evaluated as positive to 6 different fusion genes, namely t(9;22)/BCR-ABL1, t(12;21)/ETV6-RUNX1, t(4;11)/KMT2A-AFF1, del(X)/P2RY8-CRLF2, t(1;19)/TCF3-PBX, and t(9;11)/KMT2A-MLLT3, by standard methods. Our method distinguished all 6 sample-specific fusion genes within the dataset. In addition, we analyzed the same training dataset through Illumina BaseSpace, STAR-Fusion,^21^ and the customized pipeline described by Jennifer L. Winters et al.^22^ The STAR-Fusion tool did not detected 1 out of 6 fusion genes (del(X)/P2RY8-CRLF2), while the Illumina BaseSpace did not detect 2 out of 6 fusion genes (t(9;11)/KMT2A-MLLT3 and t(4;11)/ KMT2A- AFF1). The method described by Jennifer L. Winters et al. did not detect 3 out of 6 fusion genes (t(1;19)/TCF3-PBX, t(9;11)/KMT2A-MLLT3, and del(X)/P2RY8-CRLF2) (Table 1).

**Table 1:**
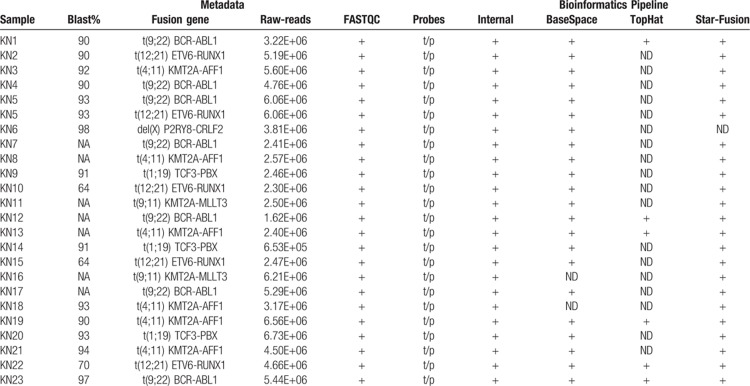
Comparison of available bioinformatics pipelines.

**FIGURE 1:**
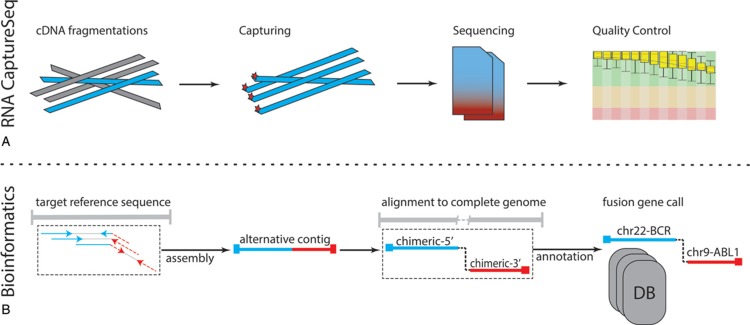
The standard operating procedure: (A) RNA CaptureSeq protocol allows the isolation of specific genomic regions (targets) through complementary probes; then, the captured fragments are sequenced, and the FASTQ file quality is evaluated. (B) The bioinformatics pipeline includes four sequential steps, which allows the identification of fusion genes through the identification of putative break-points on the genomic sequences of targeted genes.

The ability of our procedure to detect all fusion transcripts derives from the fine-tuning of the bioinformatics pipeline to cover the specific RNA target–capture scenario, where both genes involved in the fusion are not always captured (see Material and Methods and Fig. 1). For these reasons, we applied only our method in the subsequent analyses.

### Evaluation of the OP in clinical diagnosis

RNA material obtained from patient bone marrow mononuclear cells at the onset or relapse of the disease was sequenced using the RNA PanCancer (Illumina, San Diego, CA). Raw FASTQ files underwent quality control and were afterwards analyzed through our system. A detailed description of the OP strategy is available in the Materials and Methods section. The time required for the procedure from library preparation to obtaining results was 2.5 days.

We screened a cohort of 89 samples of B-cell ALL leukemia (test set) for positivity to fusion genes. All samples were negative for the fusion genes t(12;21)/ETV6-RUNX1, t(9;22)/BCR-ABL1, t(4;11)/KMT2A-AFF1, and t(1;19)*/*TCF3-PBX1 by the standard screening methods. The test set was divided into 3 groups: frontline high-risk (HR), relapse (RL), and patients with a high value of minimal residual disease (MRD) at day 33 of chemotherapy induction (TP1+). Overall, the OP identified 26 different fusion genes in 38 out of the 89 investigated samples, with the transcripts of 16 of them being of prognostic value (Table 2 and Suppl. Table 1, Supplemental Digital Content). New fusion genes in B-cell ALL and not recorded in public databases were validated through reverse transcription PCR (RT-PCR) or FISH to discern between false and true positives (Supplementary Table 2, Supplemental Digital Content).

**Table 2:**
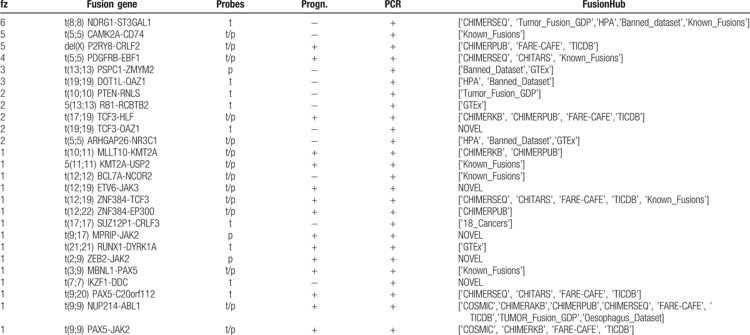
RNAseq Fusion transcripts identified by our OP.

### OP applied to the frontline HR group

**FIGURE 2:**
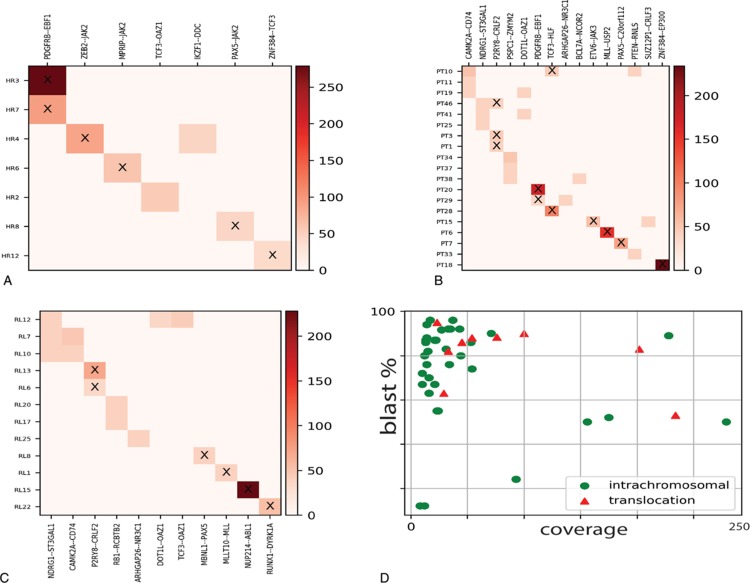
(A), (B), and (C) Heatmaps of detected fusion genes among different risk groups. The axes correspond to the detected fusion genes (X) and sample names (Y). The color code represents the coverage on the fusion gene breakpoint as reported by the scale on the right. The ‘X’ tag highlights fusion genes of prognostics relevance. (D) Fusion genes distribution in terms of intrachromosomal (green dots) or interchromosomal translocations (red triangles) in relations to the breakpoint read coverage and percentage of blast cells.

Seven out of 16 samples (43%) resulted as positive for fusion genes (Fig. 2a). Four samples carried fusion genes recurrently associated to B-cell ALL: t(5;5)/EBF1-PDGFRB (n = 2), t(9;9)/PAX5-JAK2 (n = 1), and t(12;19)*/*ZNF384-TCF3 (n = 1) and 3 samples were positive for t(19;19)/TCF3-OAZ1 (n = 1), t(7;7)/IKZF1-DDC (n = 1), t(2;9)/ZEB2-JAK2 (n = 1), and t(9;17)/MPRIP-JAK2 (n = 1) fusion genes. All fusion transcripts were confirmed by RT-PCR, while the novel fusion genes t(2;9)/ZEB2-JAK2 (n = 1) and t(9;17)/MPRIP-JAK2 were validated through FISH (Suppl. Fig. 1, Supplemental Digital Content).

### OP applied to the TP1+ group

The OP identified fusion genes in 19 out of 49 samples (38.8%) (Fig. 2b). Nine samples were evaluated as positive for fusion genes that are frequent in B-cell ALL: t(17;19)/TCF3-HLF (n = 2), del(X)/P2RY8-CRLF2 (n = 3), t(5;5)/EBF1-PDGFRB (N = 2), t(12;19)/ETV6-JAK3 (n = 1), t(12;22)/ZNF384-EP300 (n = 1). We also identified a novel inter-chromosomal rearrangement, t(9;20)/PAX5-C20orf112 (n = 1), and a variety of intra-chromosomal fusion genes (n = 9) that were already annotated in public databases, and we validated them by RT-PCR (Suppl. Table 1, Supplemental Digital Content).

### OP applied to the RL group

The OP identified fusion genes in 12 out of 24 samples of the RL group (∼50%) (Fig. 2c): t(9;9)/NUP214-ABL1 (n = 1), del(X)/P2RY8-CRLF2 (n = 2), t(10;11)/MLLT10-KMT2A (n = 1), t(21;21)/RUNX1-DYRK1A (n = 1), and t(3;9)/PAX5-MBLN1 (n = 1) fusion genes were associated with ALL and of clinical relevance for the patients and were hence immediately validated by RT-PCR. On the other hand, the OP identified additional fusion genes derived from intra-chromosomal rearrangements, such as t(8;8)/NDRG1-ST3GAL1 (n = 3), t(13;13)/RB1-RCBTB2 (n = 2), t(19;19)/DOT1L-OAZ1 (n = 1), t(19;19)/TCF3-OAZ1 (n = 1), t(5;5)/ARHGAP26-NR3C1 (n = 1), and t(5;5)/CAMK2A-CD74 (n = 2), which were already annotated in public databases.

### Enrichment of intra-chromosomal fusion genes

**FIGURE 3:**
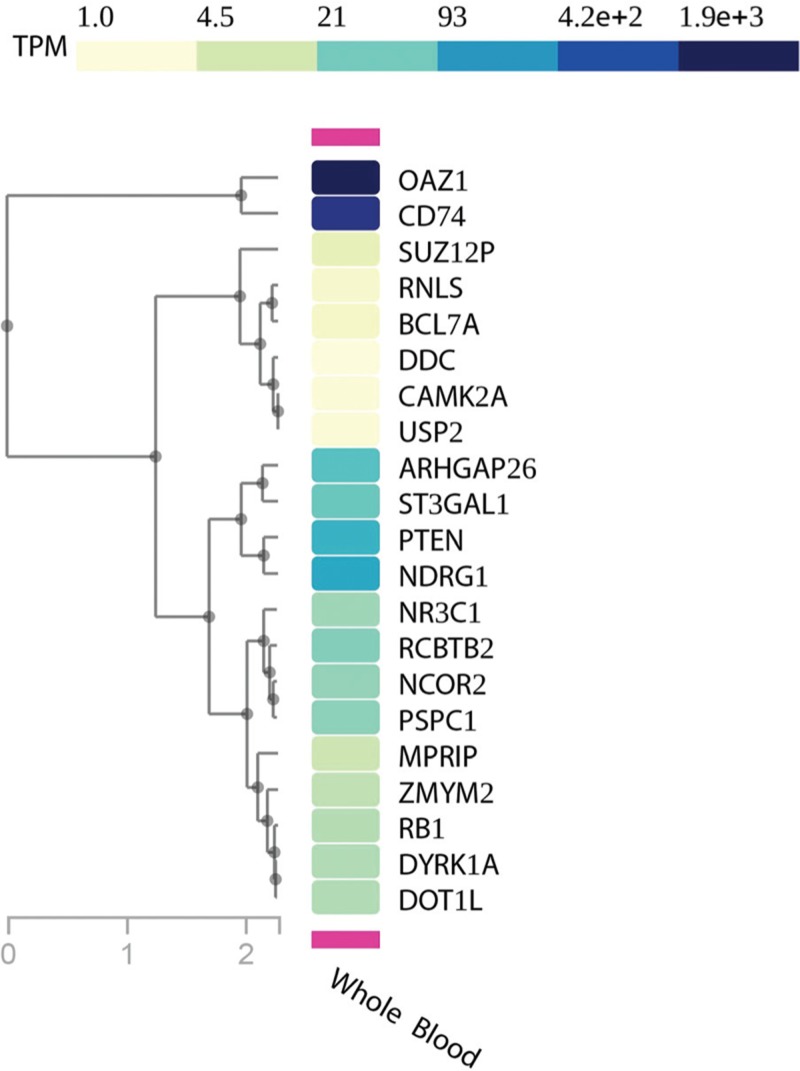
Gene expression profile of genes involved in intra-chromosomal fusion genes but not associated to ALL.

The OP identified 26 fusion genes in 38 investigated patients (HR, RL, and TP1+ groups). Among them, 17 (65%) fusion genes derived from intra-chromosomal rearrangements and were supported by a low read coverage (∼20× to ∼50×) in coexistence with high levels of blast cells in the BM (∼70% to ∼96%) (Fig. 2d). We did not observe a correlation between intra-chromosomal fusion genes associated with recurrent chromosomal translocations in B-cell ALL (Table 3). RT-PCR confirmed frequent B-cell ALL intra-chromosomal fusion genes, such as PDGFRB-EBF1, NUP214-ABL1, and PAX5-JAK2 (Suppl. Table 2, Supplemental Digital Content). P2RY8-CRLF2 fusions were not confirmed by RT-PCR since those samples correlated with del(X)(p22p22) detected by multiplex ligation-dependent probe amplification and highly expressed CRLF2 detected by gene expression profile (data not presented). We further investigated gene expression levels in healthy whole-blood samples for genes involved in intra-chromosomic fusions as well as those not known in B-cell ALL (n = 21, gene set) through the GTEx portal.^23^ Sixteen genes had transcript per million (TPM) expression levels from medium to high (TPM greater than 5.4), while 5 of them had low levels (TPM between 1 and 5.4) (Fig. 3). Also, some intra-chromosome fusion transcripts involved genes spatially close, within a range of 150 to 250 kb, and annotated as conjoined genes. Indeed, we validated those fusion gene events by RT-PCR and confirmed their nucleotide sequences by Sanger sequencing (Suppl. Table 2, Supplemental Digital Content).

**Table 3:**
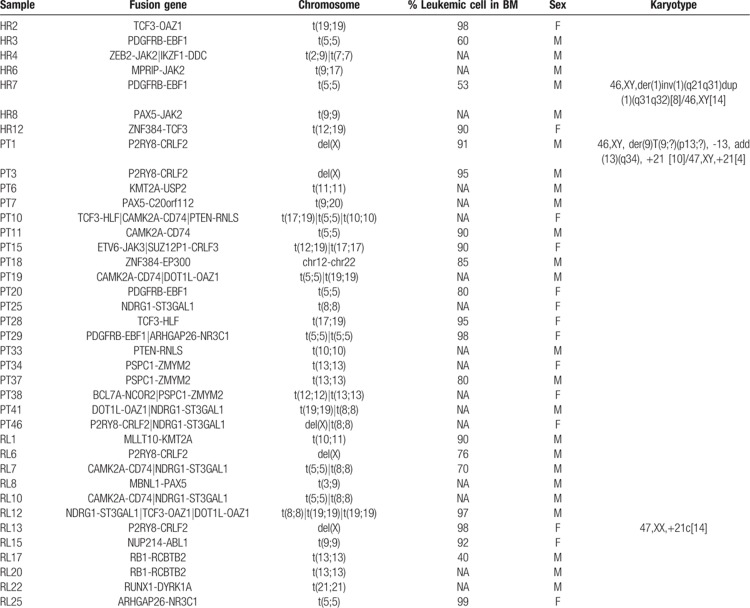
Sample-specific fusion transcripts.

## Discussion

Fusion genes are hallmarks of ALL both in pediatric and adult patients; their identification is crucial to design a risk-reducing-driven chemotherapy treatment (precision medicine). Precision medicine allows either very low-risk patients to proceed with standard therapy or very high-risk patients to be candidates for experimental and/or targeted therapies. For this purpose, sensitive, specific, and comprehensive screening of selected genomic regions prone to chromosomic breaks are needed in routine diagnostics to identify the increasing variety of fusion genes.

We built a versatile and straightforward OP to recognize fusion genes at nucleotide resolution without any a priori knowledge, which overcomes the limitations of qPCR and FISH. The OP employs an RNA CaptureSeq panel that allows targeted transcriptome sequencing through a simple library preparation protocol. For the subsequent data analysis, we fine-tuned a bioinformatics pipeline that deploys robust and stable tools, which can be easily set up on any operative system through the Anaconda Platform. Our bioinformatics pipeline recognized all fusion genes harbored by samples within the training dataset, while the Star-Fusion, Illumina BaseSpace, and the strategy proposed by Winter et al reached 83%, 66%, and 50% success in fusion transcripts identification, respectively. Prognostically significant and frequent B-cell precursor ALL fusion genes such as *KMT2A* rearrangements and P2RY8-CRLF2 were not fully detected by the external tools. Patients harboring *KMT2A* rearrangements have a particularly unfavorable prognosis.^10,24,25^*KMT2A* is prone to breaks in various genomic location with several partners, thus making the detection of its resulting fusion genes challenging. On the other hand, the repetitive nature of the chromosome X may compromise read alignment and the identification of the P2RY8-CRLF2 fusion gene. Our results indicated that our purpose-built, disease- and NGS-strategy specific bioinformatics pipeline is required for covering many possible scenarios causing fusion genes. The evaluation of the OP through the analysis of 89 pediatric B-cell precursor ALL samples identified 26 different fusion genes among 38 samples that were undetectable by the standard routine diagnostics. Sixteen of those fusion transcripts have prognostic value since they involved rearrangements in genes driving leukemogenesis (*KMT2A*, *JAK2*, and *PAX5*). Moreover, the newly identified fusion genes t(2;9)/ZEB2-JAK2 and t(9;17)/MPRIP-JAK2, which are possibly targetable by JAK/STAT inhibitors, highlight the potential of our OP for precision medicine and biomarker discovery. Additionally, we detected a case of NUP214/ABL1 fusion genes in B-cell ALL, which only 2 cases were previously reported.^26^ We confirmed the increased capability provided by RNA CaptureSeq to detect small local structural variants through the identification of a variety of intra-chromosomal fusion genes (n = 17). Multiple intra-chromosomal fusion genes were the only detected in the sample within our set of genes (n = 1385); hence, it is not possible to state any functional correlation between those rearrangements and the recurrent fusion genes (such as BCR-ABL1, ETV6-RUNX1, and *KMT2A* rearrangements). Some intra-chromosomal fusion transcripts, namely PSPC1-ZMYM2, DOT1L-OAZ1, RB1-RCBTB2, ARHGAP26-NR3C1, were also observed in NGS studies^27,28,29^ of healthy populations (e.g., GTEx, Banned_dataset, and HPA), or annotated as conjoined genes.^30,31^ We also detected intra-chromosomal fusion transcripts involving recurrent leukemogenic genes (IKZF1-DDC, P2RY8-CRLF2, KMT2A-UPS2, MLLT10-KMT2A) that are prone to deletions and with a prognostic value (such as IKZF1,^32^ and KMT2A^33^). Despite RNA CaptureSeq cannot discerns between inter- and intra- chromosome fusion genes when the same chromosomes are involved, these previous studies suggested an intra-chromosome origin.

In conclusion, herein we have described an NGS-based approach suitable for the detection of fusion genes, regardless of their expression levels, that may be incorporated into routine ALL diagnostics, with the advantage of a substantial improvement of precision medicine. Despite the OP lacks ISO certification, our finding highlights its potential and the need to develop bioinformatics tools addressing fusion genes detections from the RNA CaptureSeq scenario with precision. For this purpose, our OP may offer an idea for their implementation. Nonetheless, further studies are required to understand the biological significance and the potential therapeutic implication of the additional discoveries allowed by this tool.

## Materials and methods

### Patient cohort

A cohort of 89 B-cell precursor (BCP) ALL patients enrolled in the AIEOP-BFM ALL2009 protocol in Italy was sequenced by Illumina RNA CaptureSeq PanCancer to discern prognostic fusion genes. The cohort was composed of: 16 patients from the frontline HR group, with a level of MRD above 5 × 10^–4^ at day +78 (TP2), who were shown as fusion gene-negative during the screening; 49 patients TP1+, that is, with a high level of PCR-MRD (>5 × 10^–4^ compared to diagnostic value) at day +33 from the start of the induction therapy; and 24 patients from the RL (defined as having at least 5 × 10^–2^ blast cells after complete remission, CR). See Suppl. Table 3 (Supplemental Digital Content).

### Training dataset

A subgroup of 23 pediatric ALL patients enrolled in the AIEOP-BFM ALL2009 protocol, who were positive for fusion genes by standard clinical diagnosis, were selected. We used this subgroup as a training dataset for the development and evaluation of our bioinformatics pipeline of analysis for the assessment of fusion genes.

### FISH analysis for validating the identified fusion genes

The experiments were performed on BM metaphases from archival methanol:acetic acid-fixed chromosome suspensions, as previously described.^17^ Bacterial Artificial Chromosome (BAC) clones were opportunely selected according to the NGS data from the University of California Santa Cruz (UCSC) database (release of December 2013, GRCh38/hg38) and previously tested on normal human metaphases. Briefly, chromosome preparations from BM cells were hybridized in situ with 1 μg of each BAC probe labeled by nick translation. Hybridization was performed at 37°C in 2× saline–sodium citrate (SSC), 50% (vol/vol) formamide, 10% (w/vol) dextran sulfate, 5 μg Cot-1 DNA (Bethesda Research Laboratories, Gaithersburg, MD, USA), and 3 μg sonicated salmon sperm DNA in a volume of 10 μL. Post-hybridization washings were performed at 60°C in 0.1× SSC (3 times). In co-hybridization experiments, the probes were directly labeled with fluorescein, Cy3, and Cy5 or indirectly with biotin–dUTP and subsequently detected by 7-(diethylamino)coumarin-3-carboxylic acid *N*-succinimidyl ester-conjugated streptavidin. Chromosomes were identified by DAPI staining. Digital images were obtained using a Leica DMRXA epifluorescence microscope equipped with a cooled CCD camera (Princeton Instruments, Boston, MA). All fluorescence signals that were detected using specific filters were recorded separately as gray-scale images. Pseudo-coloring and merging of images were performed with Adobe Photoshop software.

### Enrichment analysis

Ensembl gene IDs were extracted through the BioMart API (https://www.ensembl.org/biomart). Gene expression profile data from non-diseased samples were obtained from the GTEx portal through submission of the corresponding ENSEMBL gene ID (https://gtexportal.org/home/).

### External tools for fusion gene assessment

The Illumina BaseSpace pipeline for the identification of fusion genes first aligns filtered FASTQ files to the reference human genome through the TopHat^34^ (v. 2.1.0) or STAR^35^ aligner (v. 2.5.0a). Then, the STAR aligner supports Manta-fusion and the TopHat aligner supports the TopHat-fusion^36^ to identify candidate fusion genes. For the purpose of our analysis, we required the Illumina BaseSpace to recognize the sample-specific fusion gene by at least one application. The STAR-Fusion tool, v. 1.5.0, was utilized with standard parameters on the GRCh38.p12 genome reference and the corresponding Gencode^37^ annotation set. We simulated the customized pipeline described by Jennifer L. Winters et al by deploying TopHat v. 2.1.1, which included TopHat-Fusion, and running the TopHat-Fusion pipeline with the Bowtie1^38^ flag activated.

### Operating procedure

The OP consists of a laboratory and a bioinformatics module that has been built to both maximize the efficiency and minimize the time of ALL clinical diagnostics. Each element of the laboratory module is fully customizable and commercially available, whereas each tool deployed for the bioinformatics module is freely available through the Anaconda Platform (https://www.anaconda.com/).

#### Laboratory module

##### RNA extraction protocol

Total RNA was extracted during diagnosis from bone marrow mononuclear cells by the guanidinium thiocyanate–phenol–chloroform method. Guanidine methods were used for total RNA preparation, as described by Sacchi et al.^39^

##### RNA CaptureSeq and sample sequencing

The RNA CaptureSeq ‘TruSight RNA PanCancer’ (Illumina), which includes 57,010 probes complementary to 21,043 coding regions for a total of 1385 cancer-related RNA transcripts, was applied (Fig. 1a). The protocol required 2.5 days, from library preparation to NGS sequencing. The sample libraries were prepared per the manufacturer's protocol using 10 ng of total RNA. Batches of 8 samples per run were sequenced through cartridge V3 on the Illumina MiSeq platform in a 75 bp paired-end setting for a total of 25 million paired-end reads (PE reads). The cost per sample was about 250 USD. A detailed list of targeted regions can be obtained from Illumina (https://support.illumina.com/sequencing/sequencing_kits/trusight-rna-pan-cancer-panel/downloads.html).

#### Bioinformatics module

##### FASTQ file quality control

The raw FASTQ quality control was performed using the FASTQC tool (https://www.bioinformatics.babraham.ac.uk/), which provided information on reads in terms of sequence duplication levels, per base and per sequence average quality score, sequence length distribution, and adapter content.

##### Fusion gene assessment

A purpose-built bioinformatics pipeline was developed to detect fusion genes from RNA CaptureSeq datasets. The pipeline deploys stable and open-source bioinformatics tools in a sequential mode (Fig. 1b):

– *Alignment to targets.* BWA-MEM^40^ v. 0.7.15-r1140 aligned PE reads to the genomic sequences of the targeted genes. The PE reads that did not map entirely on the reference genome through SAMTOOLS^41^ v. 1.8 were isolated; these PE reads (informative) may derive from fragments of the fusion gene breakpoint.
– *Assembly.* The informative reads are assembled into longer sequences (contigs) through the SPAdes^42^ v. 3.12.0 tool. SPAdes was run with 3 different settings of k-mer size (25, 31, and 51) to cover any possible contig scenarios, thus maximizing the sensitivity of our strategy. This step is critical since more extended sequences have a higher chance of correctly aligning on the fusion gene partner at the genomic level.
– *Alignment to the complete genome.* BWA-MEM aligned contig sequences to the complete human genome (GRCh38.p12). SAMTOOLS then retrieved contig sequences that showed chimeric features, thus mapping the 5′- and 3′-sides of different genomic locations.
– *Gene annotation and fusion gene assessment.* The chimeric sequences were annotated with BEDTOOLS^43^ v. 2.27.0 and GENCODE^37^ release 29 (GRCh38.p12) annotation. Any chimeric sequence with different gene annotation between the 5′- and 3′-side were termed fusion genes. These were queried to the web-application FusionHub^44^ to highlight fusion genes already described in other studies.
– Description of public databases is provided by the FusionHub's authors (https://www.ncbi.nlm.nih.gov/pmc/articles/PMC5929557/table/pone.0196588.t001/?report=objectonly).

## Supplementary Material

Supplemental Digital Content
