# Supplementary material for: A Simple RNA Target Capture NGS Strategy for Fusion Genes Assessment in the Diagnostics of Pediatric B-cell Acute Lymphoblastic Leukemia
Source: Hemasphere. 2019 May 22;3(3):e250. doi: 10.1097/HS9.0000000000000250 (PMC6746019; doi:10.1097/HS9.0000000000000250)

Supplementary Table 1. A detailed description of each patient and the corresponding fusion genes identified by our OP.

sample	*%blast*	*group*	*FASTQC*	*fusion gene*	*probes*	*coverage*	*progn.*	*PCR*	*Fusionhub*
HR1	71	HR	+	-		-	-	-	-
HR2	74	HR	+	t(19;19) TCF3-OAZ1	f	54	-	+	NOVEL
HR3	50	HR	+	t(5;5) PDGFRB-EBF1	f/r	279	+	+	['CHIMERSEQ', 'CHITARS', 'Known_Fusions']
HR4	-	HR	+	t(2;9) ZEB2-JAK2	r	89	+	+	NOVEL
HR4	-	HR	+	t(7;7) IKZF1-DDC	f	45	-	+	NOVEL
HR5	93	HR	+	-		-	-	-	-
HR6	-	HR	+	t(9;17) MPRIP-JAK2	r	61	+	+	NOVEL
HR7	24-Jan	HR	+	t(5;5) PDGFRB-EBF1	f/r	93	+	+	['CHIMERSEQ', 'CHITARS', 'Known_Fusions']
HR8	80	HR	+	t(9;9) PAX5-JAK2	f/r	44	+	+	['COSMIC', 'CHIMERKB', 'FARE-CAFE', 'TICDB']
HR9	90	HR	+	-		-	-	-	-
HR10	93	HR	+	-		-	-	-	-
HR11	81	HR	+	-		-	-	-	-
HR12	82	HR	+	t(12;19) ZNF384-TCF3	f/r	33	+	+	['CHIMERSEQ', 'CHITARS', 'FARE-CAFE', 'TICDB', 'Known_Fusions']
HR13	86	HR	+	-		-	-	-	-
HR14	94	HR	+	-		-	-	-	-
HR15	90	HR	+	-		-	-	-	-
HR16	96	HR	+	-		-	-	-	-

PT1	91.6	TP1+	+	del(X) P2RY8-CRLF2	f/r	27	+	+	['CHIMERPUB', 'FARE-CAFE', 'TICDB']
PT2	90	TP1+	+	-		-	-	-	-
PT3	83	TP1+	+	del(X) P2RY8-CRLF2	f/r	31	+	+	['CHIMERPUB', 'FARE-CAFE', 'TICDB']
PT4	-	TP1+	+	-		-	-	-	-
PT5	90	TP1+	+	-		-	-	-	-
PT6	50	TP1+	+	t(11;11) KMT2A-USP2	f/r	156	+	+	['Known_Fusions']
PT7	88.3	TP1+	+	t(9;20) PAX5-C20orf112	f	76	+	+	['CHIMERSEQ', 'CHITARS', 'FARE-CAFE', 'TICDB']
PT8	83	TP1+	+	-		-	-	-	-
PT9	90	TP1+	+	-		-	-	-	-
PT10	86	TP1+	+	t(17;19) TCF3-HLF	f/r	45	+	+	['CHIMERSEQ', 'CHITARS', 'FARE-CAFE', 'TICDB']
PT10	86	TP1+	+	t(5;5) CAMK2A-CD74	f/r	53	-	+	['Known_Fusions']
PT10	86	TP1+	+	t(10;10) PTEN-RNLS	f	13	-	+	['Tumor_Fusion_GDP']
PT11	87	TP1+	+	t(5;5) CAMK2A-CD74	f/r	21	-	+	['Known_Fusions']
PT12	80	TP1+	+	-		-	-	-	-
PT13	40	TP1+	+	-		-	-	-	-
PT14	79	TP1+	+	-		-	-	-	-
PT15	88	TP1+	+	t(12;19) ETV6-JAK3	f/r	54	+	+	NOVEL
PT15	88	TP1+	+	t(17;17) SUZ12P1-CRLF3	f	14	-	+	['18_Cancers']
PT16	86.6	TP1+	+	-		-	-	-	-

PT17	55	TP1+	+	-		-	-	-	-
PT18	53	TP1+	+	t(12;22) ZNF384-EP300	f/r	234	+	+	['CHIMERPUB']
PT19	80	TP1+	+	t(5;5) CAMK2A-CD74	f/r	12	-	+	['Known_Fusions']
PT19	80	TP1+	+	t(19;19) DOT1L-OAZ1	f	12	-	+	['HPA', 'Banned_Dataset']
PT20	52	TP1+	+	t(5;5) PDGFRB-EBF1	f/r	175	+	+	['CHIMERSEQ', 'CHITARS', 'Known_Fusions']
PT21	83	TP1+	+	-		-	-	-	-
PT22	90	TP1+	+	-		-	-	-	-
PT23	82	TP1+	+	-		-	-	-	-
PT24	36	TP1+	+	-		-	-	-	-
PT25	76	TP1+	+	t(8;8) NDRG1-ST3GAL1	f	14	-	+	['CHIMERSEQ', 'Tumor_Fusion_GDP','HPA','Banned_dataset','Known_Fusions']
PT26	81	TP1+	+	-		-	-	-	-
PT27	96	TP1+	+	-		-	-	-	-
PT28	90	TP1+	+	t(17;19) TCF3-HLF	f/r	100	+	+	['CHIMERKB', 'CHIMERPUB', 'FARE-CAFE','TICDB']
PT29	96	TP1+	+	t(5;5) PDGFRB-EBF1	f/r	37	+	+	['CHIMERSEQ', 'CHITARS', 'Known_Fusions']
PT29	96	TP1+	+	t(5;5) ARHGAP26-NR3C1	f/r	19	-	+	['HPA', 'Banned_Dataset','GTEx']
PT30	87	TP1+	+	-		-	-	-	-
PT31	56	TP1+	+	-		-	-	-	-
PT32	90	TP1+	+	-		-	-	-	-
PT33	70	TP1+	+	t(10;10) PTEN-RNLS	f	16	-	+	['Tumor_Fusion_GDP']

PT34	82	TP1+	+	t(13;13) PSPC1-ZMYM2	r	15	-	+	['Banned_Dataset','GTEx']
PT35	80	TP1+	+	-		-	-	-	-
PT36	90	TP1+	+	-		-	-	-	-
PT37	72	TP1+	+	t(13;13) PSPC1-ZMYM2	r	10	-	+	['Banned_Dataset','GTEx']
PT38	67	TP1+	+	t(12;12) BCL7A-NCOR2	f/r	21	-	+	['Known_Fusions']
PT38	67	TP1+	+	t(13;13) PSPC1-ZMYM2	r	10	-	+	['Banned_Dataset','GTEx']
PT39	82	TP1+	+	-		-	-	-	-
PT40	76	TP1+	+	-		-	-	-	-
PT41	63	TP1+	+	t(19;19) DOT1L-OAZ1	f	16	-	+	['HPA', 'Banned_Dataset']
PT41	63	TP1+	+	t(8;8) NDRG1-ST3GAL1	f	16	-	+	['CHIMERSEQ', 'Tumor_Fusion_GDP','HPA','Banned_dataset','Known_Fusions']
PT42	45	TP1+	+	-		-	-	-	-
PT43	52	TP1+	+	-		-	-	-	-
PT44	80	TP1+	+	-		-	-	-	-
PT45	86	TP1+	+	-		-	-	-	-
PT46	55	TP1+	+	del(X) P2RY8-CRLF2	f/r	24	+	+	['CHIMERPUB', 'FARE-CAFE', 'TICDB']
PT46	55	TP1+	+	t(8;8) NDRG1-ST3GAL1	f	23	-	+	['CHIMERSEQ', 'Tumor_Fusion_GDP','HPA','Banned_dataset','Known_Fusions']
PT47	86	TP1+	+	-		-	-	-	-
PT48	78	TP1+	+	-		-	-	-	-
PT49	92	TP1+	+	-		-	-	-	-

RL1	63	RL	+	t(10;11) MLLT10-KMT2A	f/r	29	+	+	['CHIMERKB', 'CHIMERPUB']
RL2	48	RL	+	-		-	-	-	-
RL3	80	RL	+	-		-	-	-	-
RL4	48	RL	-	-		-	-	-	-
RL5	76	RL	+	-		-	-	-	-
RL6	76	RL	+	del(X) P2RY8-CRLF2	f/r	34	+	+	['CHIMERPUB', 'FARE-CAFE', 'TICDB']
RL7	65	RL	+	t(5;5) CAMKA2A-CD74	f/r	47	-	+	NOVEL
RL7	65	RL	+	t(8;8) NDRG1-ST3GAL1	f	30	-	+	['CHIMERSEQ', 'Tumor_Fusion_GDP','HPA','Banned_dataset','Known_Fusions']
RL8	95	RL	+	t(3;9) MBNL1-PAX5	f/r	23	+	+	['Known_Fusions']
RL9	94	RL	+	-		-	-	-	-
RL10	87	RL	+	t(5;5) CAMK2A-CD74	f/r	13	-	+	['Known_Fusions']
RL10	87	RL	+	t(8;8) NDRG1-ST3GAL1	f	22	-	+	['CHIMERSEQ', 'Tumor_Fusion_GDP','HPA','Banned_dataset','Known_Fusions']
RL11	89	RL	+	-		-	-	-	-
RL12	92	RL	+	t(8;8) NDRG1-ST3GAL1	f	33	-	+	['CHIMERSEQ', 'Tumor_Fusion_GDP','HPA','Banned_dataset','Known_Fusions']
RL12	92	RL	+	t(19;19) TCF3-OAZ1	f	43	-	+	NOVEL
RL12	92	RL	+	t(19;19) DOT1L-OAZ1	f	35	-	+	['HPA', 'Banned_Dataset']

RL13	90	RL	+	del(X) P2RY8-CRLF2	f/r	71	+	+	['CHIMERPUB', 'FARE-CAFE', 'TICDB']
RL15	89	RL	+	t(9;9) NUP214-ABL1	f/r	228	+	+	['COSMIC','CHIMERAKB','CHIMERPUB','CHIMERSEQ', 'FARE-CAFE', 'TICDB','TUMOR_Fusion_GDP','Oesophagus_Dataset]
RL16	60	RL	-	-		-	-	-	-
RL17	12	RL	+	t(13;13) RB1-RCBTB2	f	8	-	+	['GTEx']
RL18	12	RL	+	-		-	-	-	-
RL19	96	RL	+	-		-	-	-	-
RL20	12	RL	+	t(13;13) RB1-RCBTB2	f	12	-	+	['GTEx']
RL21	65	RL	+	-		-	-	-	-
RL22	85	RL	+	t(21;21) RUNX1-DYRK1A	f	54	+	+	['GTEx']
RL23	83	RL	+	-		-	-	-	-
RL24	-	RL	+	-		-	-	-	-
RL25	94	RL	+	t(5;5) ARHGAP26-NR3C1	f/r	14	-	+	['HPA', 'Banned_Dataset','GTEx']

Supplementary Table 2: RT-PCR forward and reverse primer sequences for the validated fusion transcripts, and the corresponding Sanger Sequencing File Name.

fusion genes	*forward*	*5'-3' sequence*	*reverse*	*5'-3' sequence*	*product size*	*File Name (.ab)*
t(11;11) KMT2A-USP2	MLLex19_F	AAGGTCCTGGAGAACCAGACT	USP2ex5_R	AGATGGGCTCACCACATCATT	701	MLL-USP2_FOR_52CF79;
t(12;19) ETV6-JAK3	ETV6 ex3_F	GGAGCAGGGATGACGTAGC	JAK3 ex19_R	ACCAGGGCACCTGTATTGTC	487	ETV6-JAK3_FOR_93DG11;
t(12;19) ZNF384-TCF3	TCF3ex11 F	ATGGAGCAGAGGTGAACGGT	ZNF384ex6 R	TGGGGAAGGTCTGAGCTGAT	687	ZNF384-TCF3_FOR_81FI62;
t(12;22) ZNF384-EP300	EP300ex6_F	TCCCCCTCAAAAATGCTGGT	ZNF384ex4_R	ATTCTGGGTAACGGACGCTT	551	ZNF384-EP300_FOR_93DH34;
t(13;13) RB1-RCBTB2	RCBTB2ex11_F	CTGCACTGACGACGTGTTTG	RB1ex18_R	CTGCTGCAGTGTGATTATTCTGG	307	RB1-RCBTB2_FOR_98EG11;
t(17;19) TCF3-HLF	E2A-A	CACCAGCCTCATGCACAAC	HLF-1301R	GGGCCGAGTTCTCCTTCTCCAGGA	213+retained intron	TCF3-HLF_FOR_95FF26;
t(9;17) MPRIP-JAK2	JAK2ex7_F	ACGGTGGAATTCAGTGGTCAA	MPRIPex21_R	GCACTGTCAGGGGACTTCTC	412	MPRIP-JAK2_FOR_93DG07;
t(19;19) DOT1L-OAZ1	OAZ1ex1-F	CGGATGGTGAAATCCTCCCTG	DOT1Lex6-R	TGCTTTCTCGACGCCATAGT	513	DOT1L-OAZ1_ONE_20ED34;
t(19;19) TCF3-OAZ1	OAZ1ex1_F	CGGATGGTGAAATCCTCCCTG	TCF3ex6_R	TCTCCCGAAGGAGGCATAGG	343	TCF3-OAZ1_FOR_81GA68;
t(3;9) MBNL1-PAX5	PAX5_ex3_F	ACGCCAAAATCCCACCATGT	MBNL1ex4_R	CACCCGGATTCCCTGTAACC	609	PAX5-MBNL1_FOR_81FJ29;
t(5;5) ARHGAP26-NR3C1	ARHGAP26ex15-F	ACCCCAAGACTGCTTCTGAG	NR3C1ex2-R	CCCCGAGGAAAGGCTGATTT	611	ARHGAP26-NR3C1_FOR_95FF55;
t(5;5) CAMK2A-CD74	CD74ex4_F	TGCAGAATGCCACCAAGTATG	CAMK2Aex4_R	TCAGGTAGTGGTGTCCCTCC	443	CAMK2A-CD74_TWO_52CF51
t(5;5) PDGFRB-EBF1	EBF1e14F2	CACGAGCATGAACGGATACGGCTCT	PDGFRBe12R1	ATGGCCGTCAGAGCTCACAGACTCA	250	EBF1-PDGFRB_FOR_95FG05;

t(9;20) PAX5-C20orf112	PAX5_ex3 F	ACGCCAAAATCCCACCATGT	C20orf112_ex11 R	TGAGGTTTTCCAGTTCATCTGC	500	PAX5-C20ORF112_FOR_81FJ76;
t(9;9) NUP214-ABL1	NUP214ex25_F	CTACTCCACAGCCAAAACACCTCA	ABL1e4_R	GCCACCGTCAGGCTGTATTTCTTCC	696	NUP214-ABL1_FOR_98EF26;
t(9;9) PAX5-JAK2	PAX5 ex3 F	ACGCCAAAATCCCACCATGT	JAK2 ex20 R	AGCACTGTAGCACACTCCCT	622	PAX5-JAK2_FOR_81FI58-M;

Supplementary Table 3: Samples features, such as age, sex, immunophenotype, and minimal residual diseases (MRD) according to BFM protocol (Conter at el, Blood, 2010). MRD values: SR is negative at day +33 and +78 with marker sensitivity of $1x10^{-4}$; HR is positive to mrd >= $5x10^{-4}$ at day +78; SER positive to mrd >= $5x10^{-4}$ at both day+33 and day+78; MR positive to mrd <= $5x10^{-4}$ at both day+33 and day+78.

Sample	*Age*	*Sex*	*Immunophenotype*	*MRD*	*Sample*	*Age*	*Sex*	*Immunophenotype*	*MRD*
HR1	10	M	BII	HR	*PT34*	13		BII	HR
HR10	5	F	BIII	HR	*PT35*	4	M	BII	SER
HR11	16	M	BII	HR	*PT36*	15	M	BII	SER
HR12	6	F	BII	SER	*PT37*	5	M	BII	MR
HR13	18	F	BII	HR	*PT38*	4	F	BIII	MR
HR15	8	M	BII	HR	*PT4*	NA	M	NA	NA
HR16	5	F	BII	SER	*PT40*	5	F	BII	MR
HR2	10	F	BII	HR	*PT41*	10	M	BII	MR
HR3	16	F	BII	HR	*PT42*	12	M	BII	MR
HR4	NA	M	NA	NA	*PT43*	14	F	BII	MR
HR5	14	M	BII	SER	*PT44*	NA	M	NA	NA
HR6	18	M	BII	NA	*PT45*	4	M	BII	SER
HR7	16	M	BIII	HR	*PT46*	5	F	BII	SER
HR8	5	M	BIII	MR	*PT47*	7	M	BII	HR
HR9	14	F	BII	HR	*PT48*	7	F	BIII	SER
PT1	13	M	BIII	MR	*PT49*	10	F	BIII	MR
PT10	11	F	BII	HR	*PT5*	4	M	BII	HR
PT11	14	M	BII	SER	*PT6*	4	M	BII	NA
PT12	10	M	BII	HR	*PT7*	6	M	BII	HR
PT13	5	F	BIII	MR	*PT8*	11	F	BII	MR
PT14	5	F	BII	MR	*PT9*	7	F	BII	SER
PT15	7	F	BII	SER	*RL1*	10	M	BI	SR
PT16	5	F	BIII	SER	*RL10*	18	M	NA	NA
PT17	16	M	BII	SER	*RL11*	5	M	BII	SER
PT18	16	M	BII	SER	*RL12*	14	M	BII	SER
PT19	14	M	BII	HR	*RL13*	12	F	BIII	MR
PT2	14	F	BII	SER	*RL14*	14	F	BII	NO HR
PT20	14	F	BIII	MR	*RL16*	6	M	BII	MR

PT21	5	M	BII	HR	*RL17*	NA	F	NA	NA
PT22	6	M	BII	SER	*RL18*	10	F	BII	MR
PT23	6	M	BIII	SER	*RL19*	10	M	BII	HR
PT24	5	F	BIII	SER	*RL2*	13	M	BII	MR
PT25	5	F	BII	MR	*RL21*	18	M	B	MR
PT26	11	F	BII	SER	*RL22*	14	M	BII	MR
PT27	8	M	BII	MR	*RL23*	NA	F	NA	NA
PT28	5	F	BII	NA	*RL24*	6	F	NA	NA
PT29	11	F	BII	HR	*RL25*	13	F	BII	MR
PT3	7	M	BII	HR	*RL3*	18	F	BII	MR
PT30	NA	M	BII	MR	*RL5*	13	F	BII	MR
PT31	14	M	BIII	SER	*RL6*	14	M	BII	SR
PT32	7	M	BIII	HR	*RL7*	18	M	BII	HR
PT33	13	M	BIII	NA	*RL8*	NA	M	NA	NA
					RL9	5	M	BII	SR

Supplementary Figure 1: a) Fluorescence *in-situ* validation of the rearrangement between chromosome 2q22.3 and chromosome 9p24.1 causing the expressed fusion gene *ZEB2-JAK2*. B) Fluorescence *in-situ* validation of the rearrangement between chromosome 9p24.1 and chromosome 17q11.2 causing the expressed fusion gene *JAK2-MPRIP*.

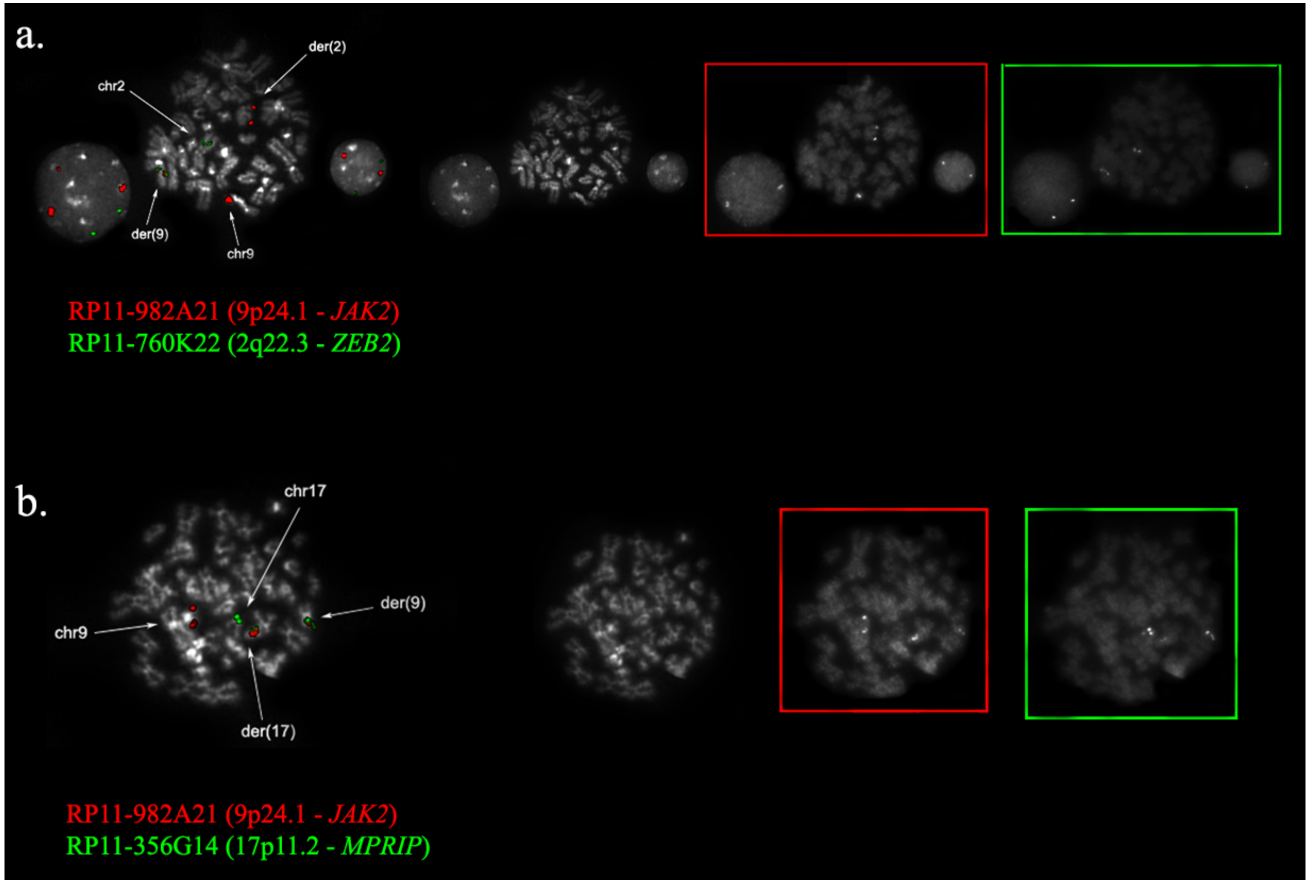

File: ARHGAP26-NR3C1_FOR_95FF55.ab1
Comment: Kundensequenzierung-EasySeq
585 bases in 7170 scans

Run Ended: Jun 20, 2017, 21:19:28
Sequence: 32617393
Page 1 of 1

Signal G:1938 A:3653 T:3514 C:4380
Lane: 4 Base spacing 14.29

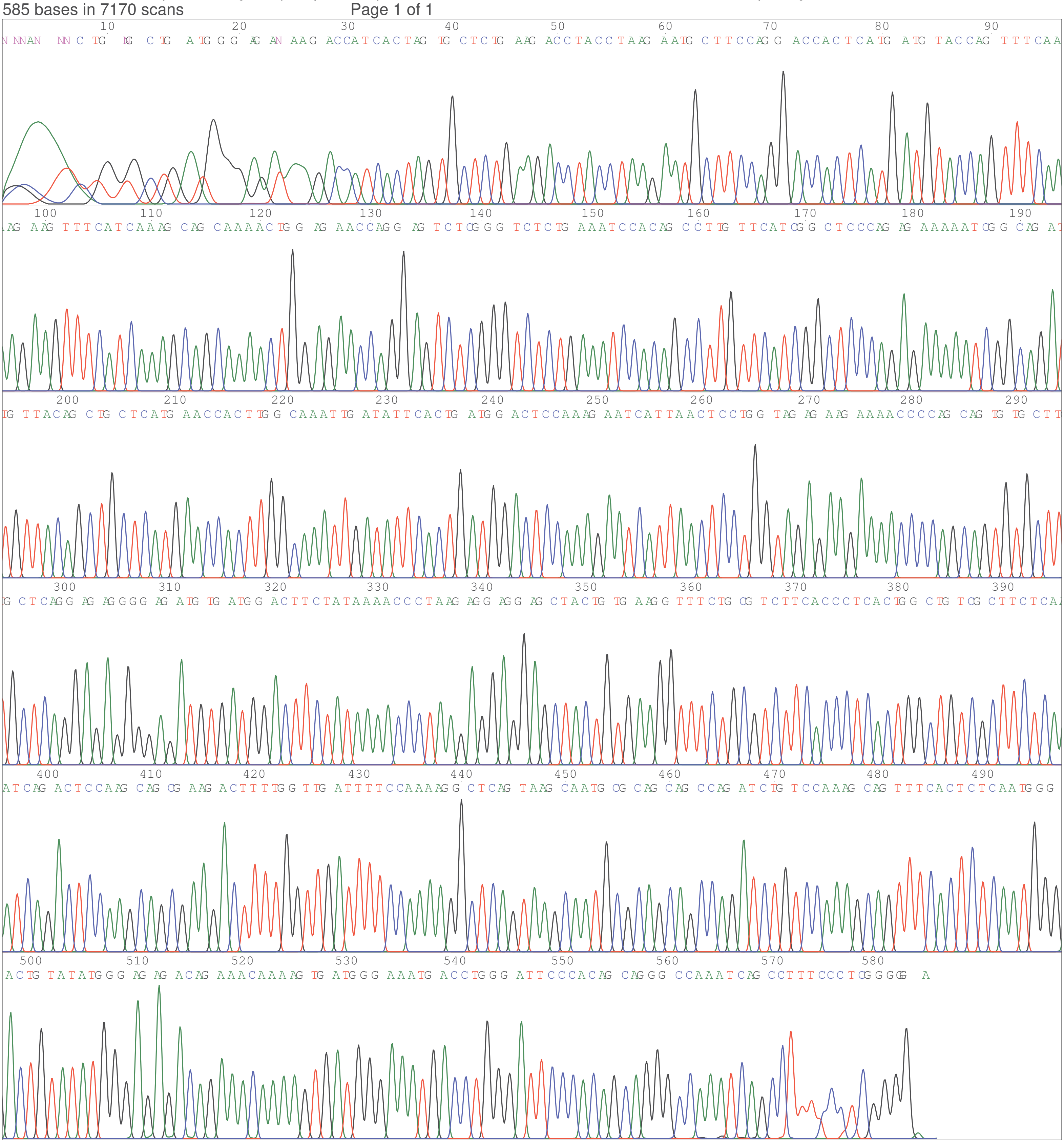

File: CAMK2A-CD74_TWO_52CF51.ab1 Run Ended: Jun 19, 2018, 5:07:44 Signal G:141 A:114 T:111 C:137
Comment: M2S00021165_48538909_F06 Sequence: 48538909 Lane: 38 Base spacing 15.20
420 bases in 16300 scans

10 20 30 40 50 60 70 80 90 100

GGGTAAAAAGGAACATTGTGTATGCAACTGCTCAGAATGCTGACCCCCTGAAGGTGTACCCGCCACTGAAGGGGAGCTTCCCGGAGAACCTGAGACACCTTAA

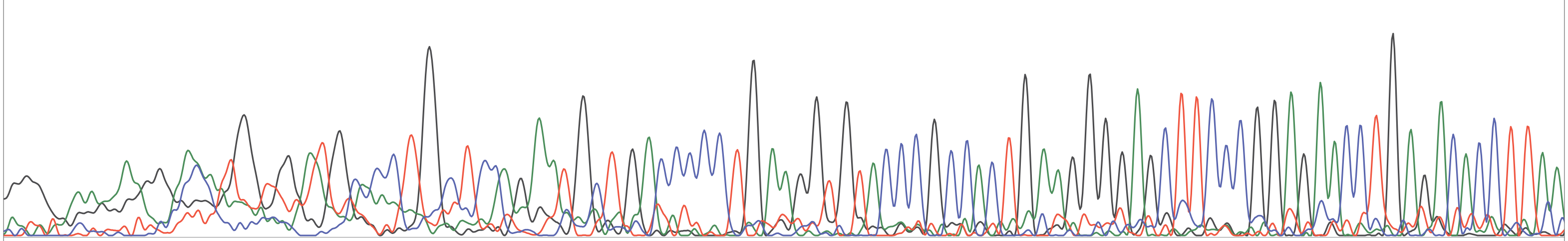

110 120 130 140 150 160 170 180 190 200

GAACACCATGGAGACCATAGACTGGAAGGTCTTTGAGAGCTGGATGCACCATTGGCTCCTGTTTGAAATGAGCAGGCACTCCTTGGAGCAAAAGCCCACTGA

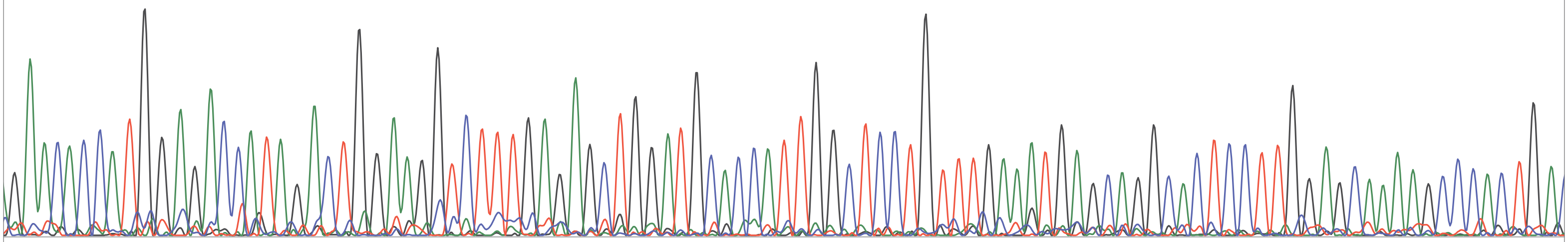

210 220 230 240 250 260 270 280 290 300

CGCTCCACCGAAAGGGGAGCCTTCTCGGTGGTGCGAAGGTGTGTGAAGGTGCTGGCTGGCCAGGAGTATGCTGCCAAGATCATCAACACAAAGAAGCTGTCAG

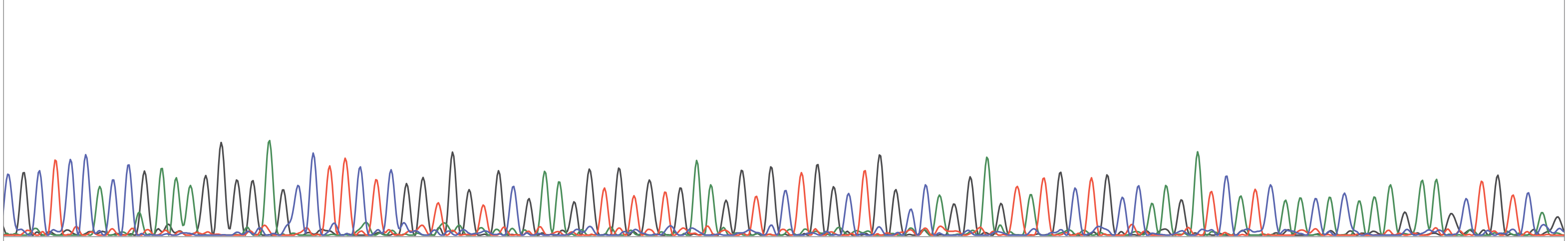

310 320 330 340 350 360 370 380 390 400 41

CCAGAGACCATCAGAAGCTGGAGCGTGAAGCCCGCATCTGCCGCCTGCTGAAGCACCCCAACATCGTCCGACTACATGACAGCATCTCAGAGGAGGGACACC

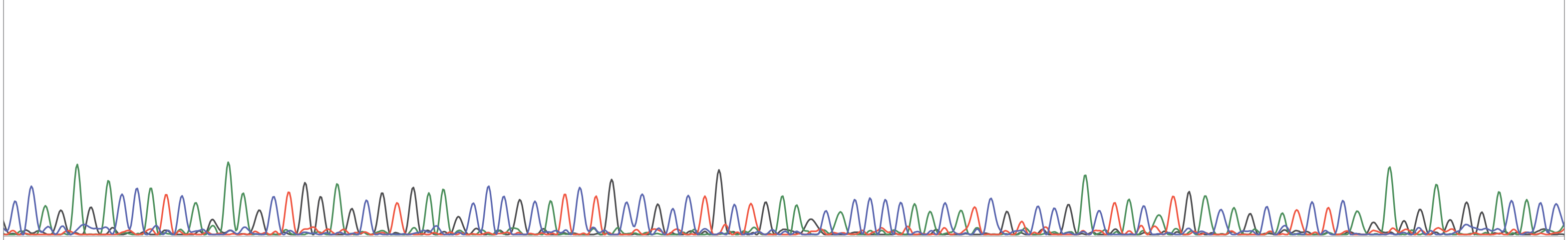

0 420

ACTACCTGAA

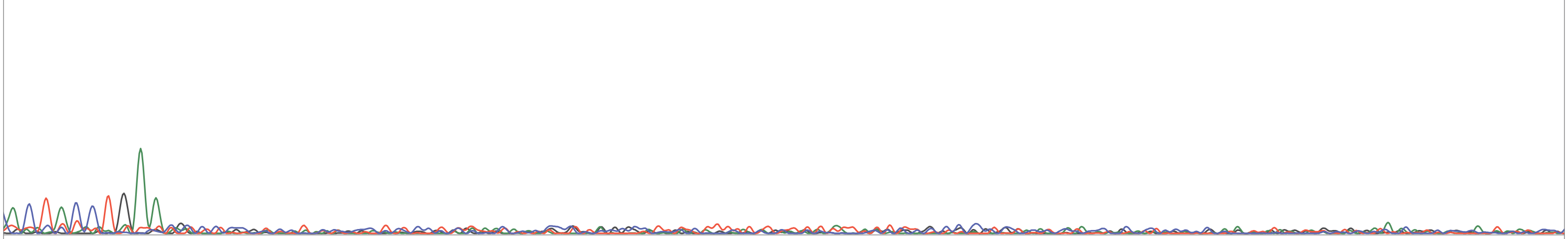

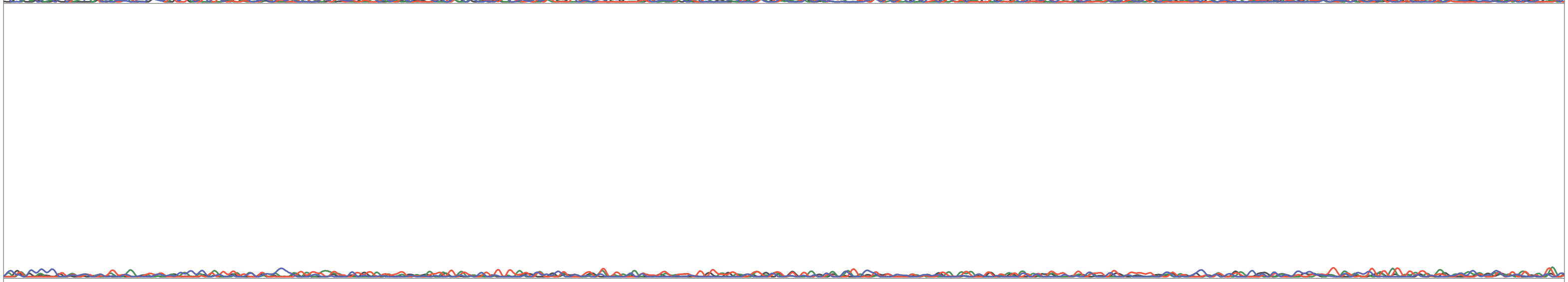

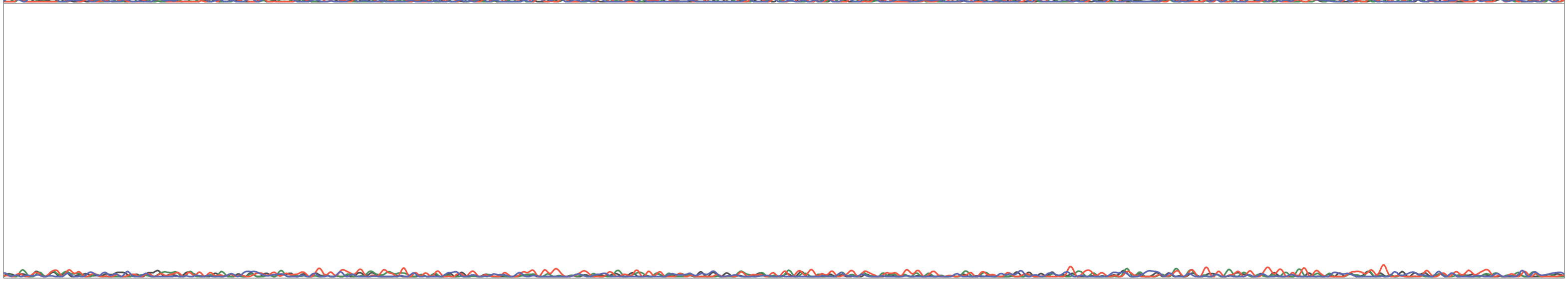

File: DOT1L-OAZ1_ONE_20ED34.ab1
Comment: Kundensequenzierung-EasySeq
608 bases in 7419 scans

Run Ended: Aug 7, 2017, 18:27:16
Sequence: 33748144

Signal G:950 A:735 T:762 C:1170
Lane: 36 Base spacing 14.32

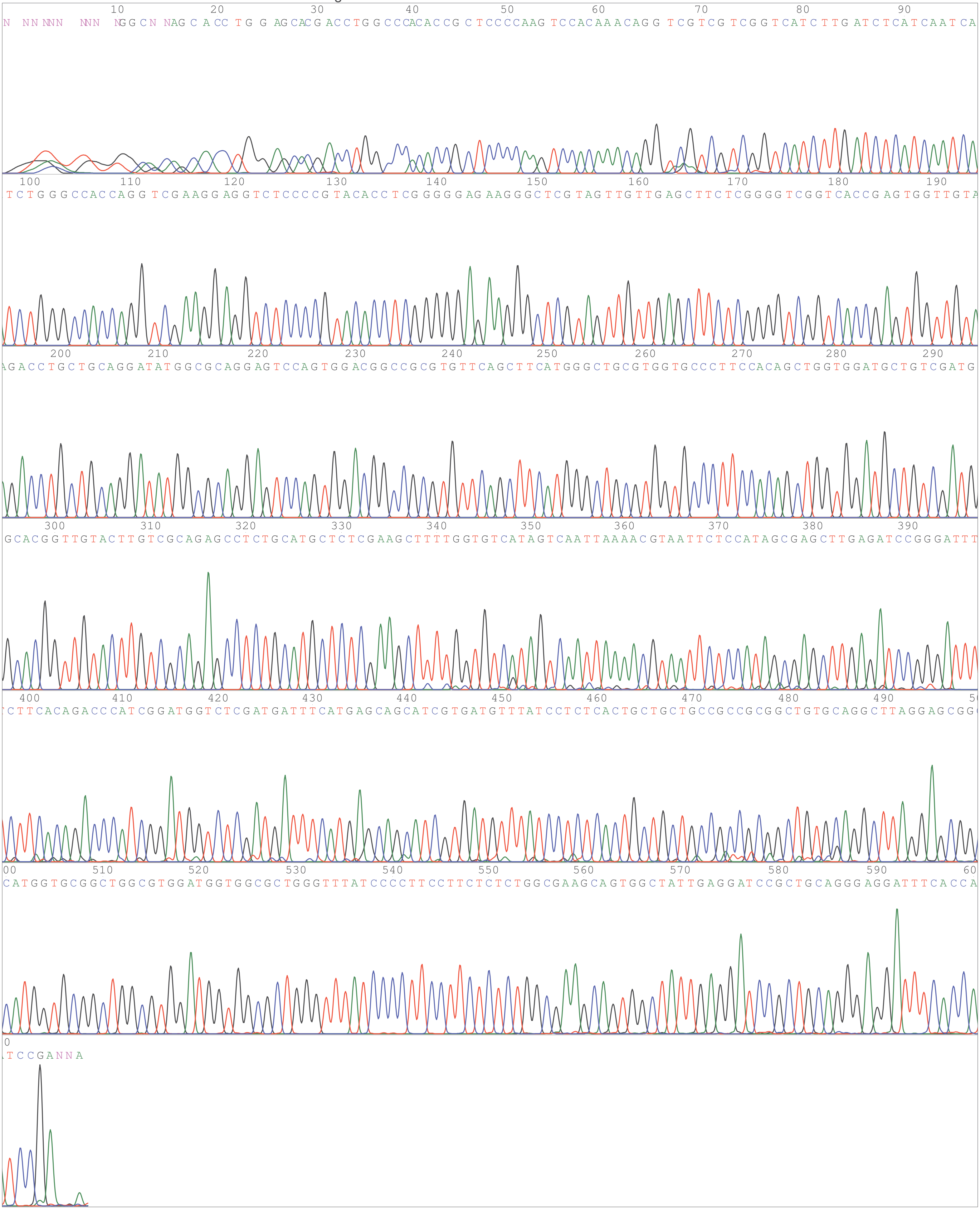

File: DOT1L-OAZ1_TWO_20ED35.ab1 Run Ended: Aug 7, 2017, 18:27:16 Signal G:451 A:372 T:381 C:542
Comment: Kundensequenzierung-EasySeq Sequence: 33748156 Lane: 59 Base spacing 14.50
612 bases in 7589 scans Page 1 of 1

10 20 30 40 50 60 70 80 90
N N N N NN NNN NNN NAN CAG C A CC T G G AG CAC G ACC T G G CCCACACCG C T CCCCAAG T CCACAAAACAGG T CG T CG T CG G T CAT C T T G A T C T CAT CAAT

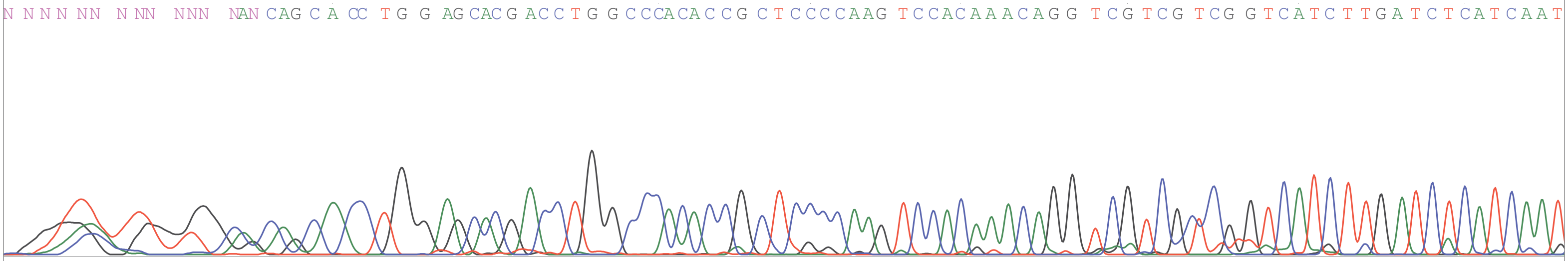

100 110 120 130 140 150 160 170 180 190
CATCTGGGCCACCAGGTCGAAGGAGGTCTCCCCGTACACCTCGGGGGAGAAGGGCTCGTAGTTGTTGAGCTTCTCGGGGTCGGTCACCGAGTGGTTG

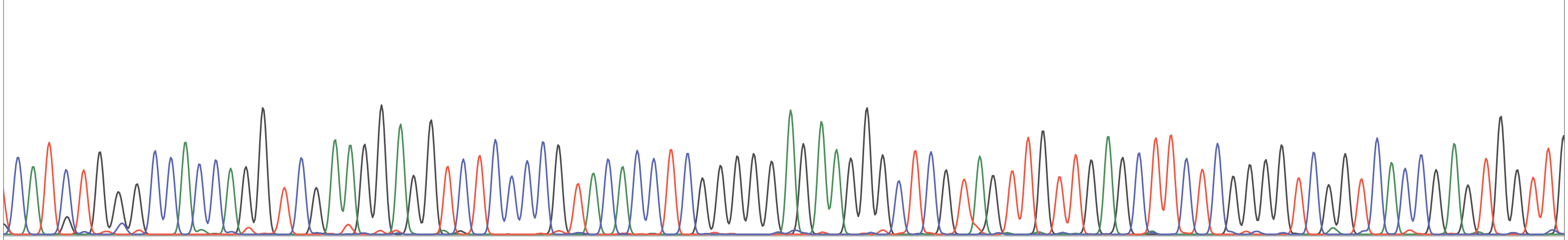

200 210 220 230 240 250 260 270 280 290
GTAGACCTGCTGCAGGATATGGCGCAGGAGTCCAGTGGACGGCCGCGTGTTCAGCTTCATGGGCTGCGTGGTGCCCTTCCACAGCTGGTGGATGCTGTCGATG

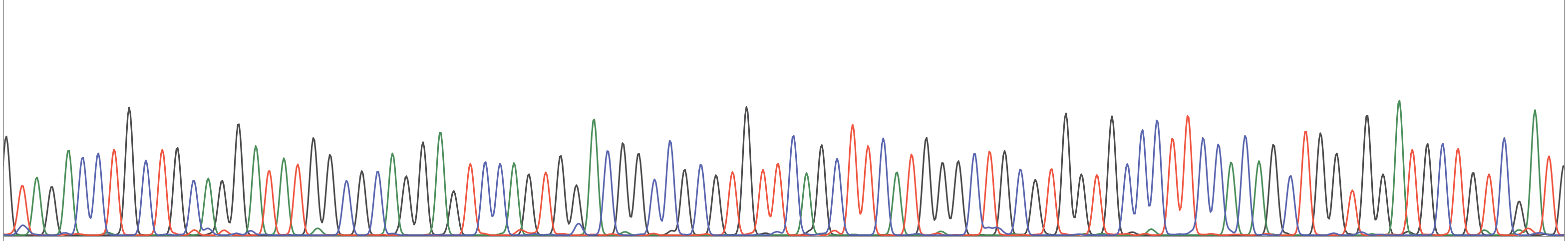

300 310 320 330 340 350 360 370 380 390
GGCACGGTTGTACTTGTCGCAGAGCCTCTGCATGCTCTCGAAGCTTTTGGTGTCATAGTCAATTAAAACGTAATTCTCCATAGCGAGCTTGAGATCCGGGATTT

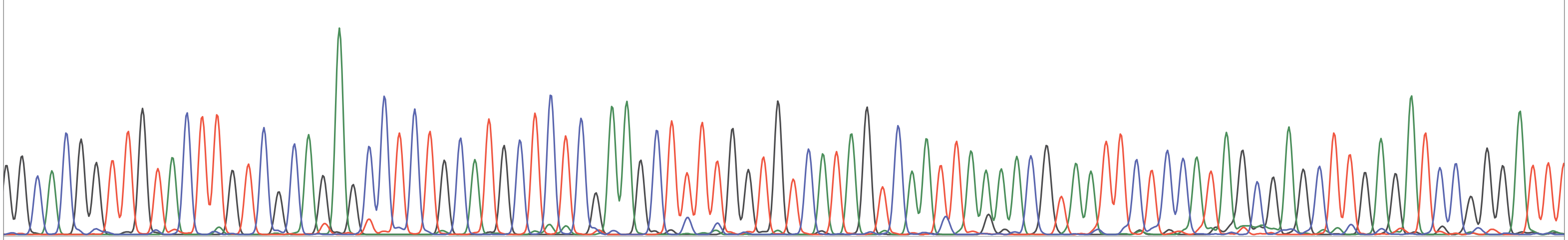

400 410 420 430 440 450 460 470 480 490
TCTTCACAGACCCATCGGATGGTCTCGATGATTTCATGAGCAGCATCGTGATGTTTATCCTCTCACTGCTGCTGCCGCCGCGGCTGTGCAGGCTTAGGAG

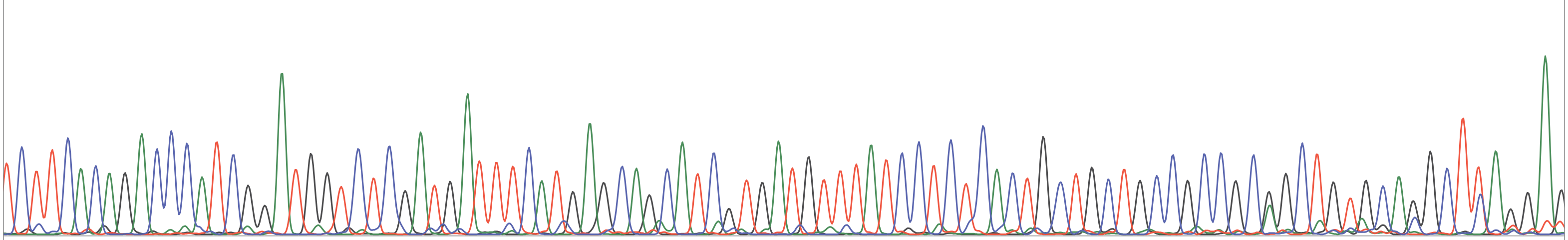

500 510 520 530 540 550 560 570 580 590
GCGGCATGGTGCGGCTGGCGTGGATGGTGGC GCTGGGTTTATCCCCTTCCTTCTCTCTGG TGAAGCAGTGGCTATTGTAGGATCCTGCTG CAGG

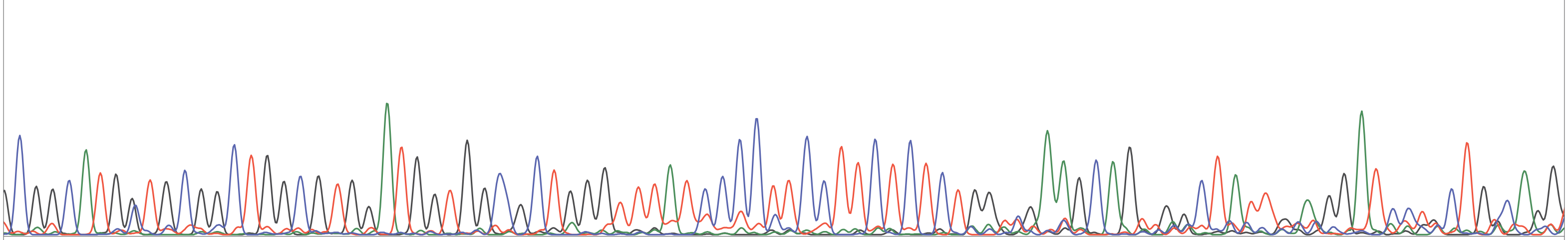

600 610
T NAGGGATTTCTTCCATCCGA G

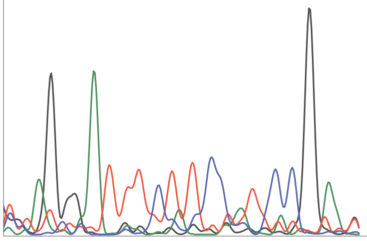

File: EBF1-PDGFRB_FOR_95FG05.ab1
Comment: Kundensequenzierung-EasySeq
220 bases in 2758 scans

Run Ended: Jul 13, 2017, 1:41:29
Sequence: 33166162
Page 1 of 1

Signal G:1472 A:1671 T:2538 C:3452
Lane: 75 Base spacing 14.83

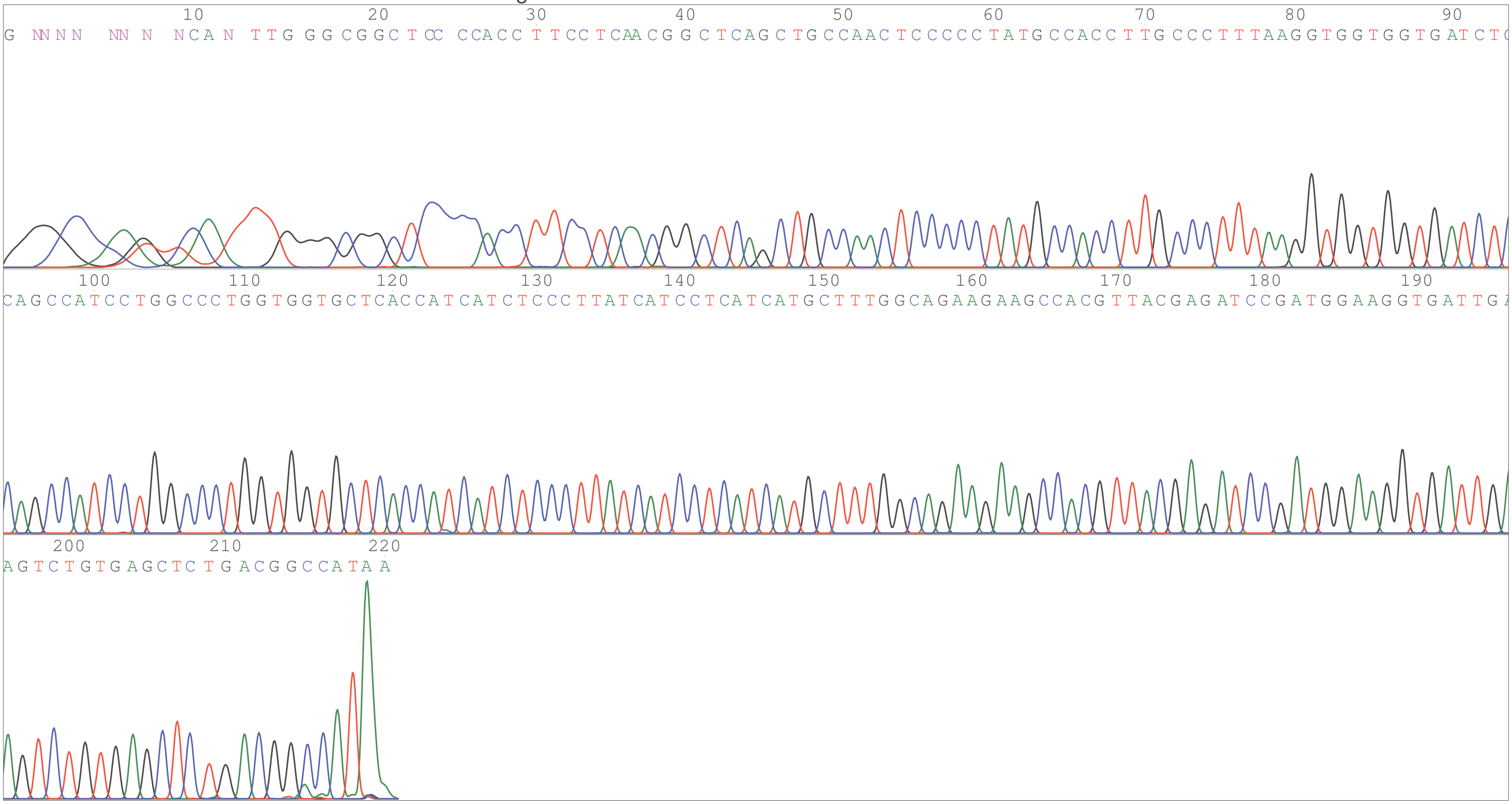

File: ETV6-JAK3_FOR_93DG11.ab1
Comment: Kundensequenzierung-EasySeq
457 bases in 5579 scans

Run Ended: Jan 17, 2018, 20:11:56
Sequence: 37189494
Page 1 of 1

Signal G:461 A:433 T:448 C:603
Lane: 40 Base spacing 13.70

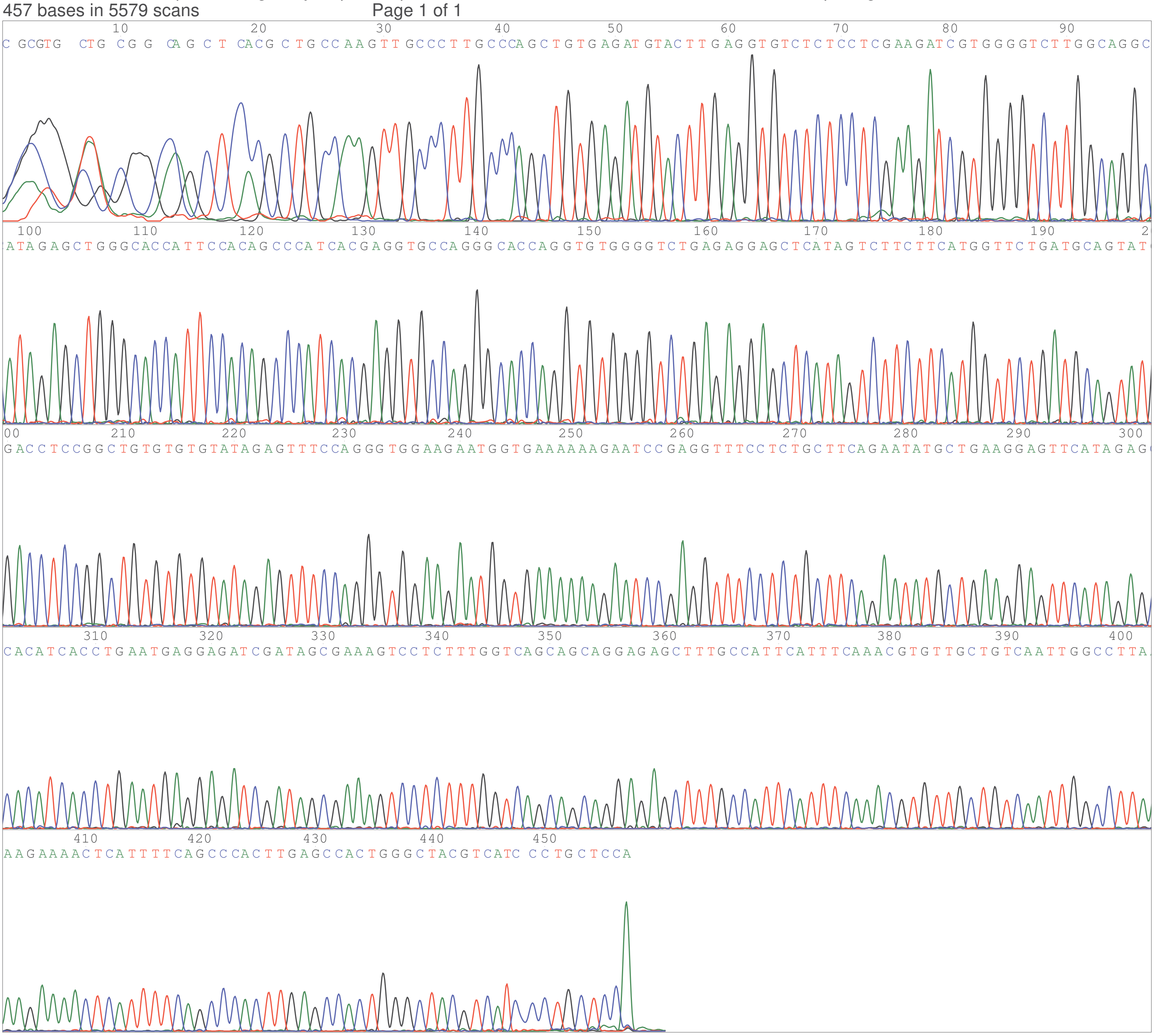

File: MLL-USP2_FOR_52CF79.ab1 | Run Ended: Jun 28, 2018, 11:53:44 | Signal G:1169 A:1283 T:1439 C:1456
Comment: M2S00021943_48539180_E10 | Sequence: 48539180 | Lane: 72 Base spacing 16.40
711 bases in 19655 scans |

10 20 30 40 50 60 70 80 90 10
ACCC ATC TT CATC TCC TACACCACCAATTTTGAGTACTGATAGGAGTCGAGAAGACAGTCCAGAGCTGAACCCACCCCCAGGCATAGAAGACAATAGACA

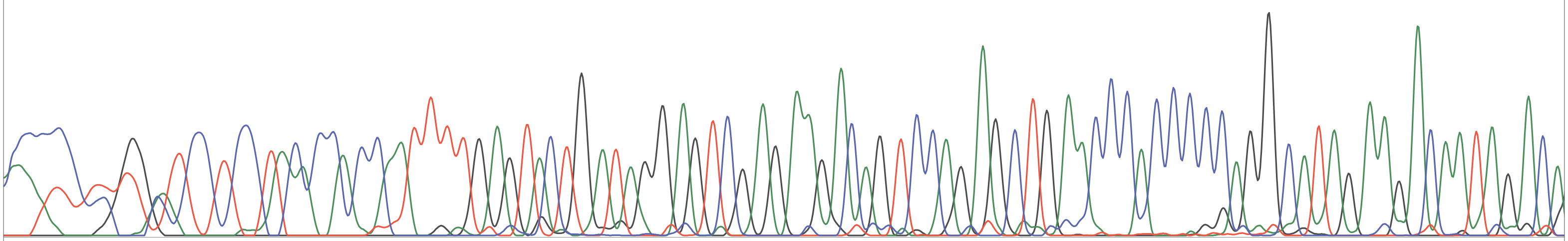

0 110 120 130 140 150 160 170 180 190
GTGTGCGTTATGTTTGACTTATGGTGATGACAGTGCTAATGATGCTGGTCGTTTACTATATATTGGCCAAAATGAGTGGACACATGTAAATTGTGCT

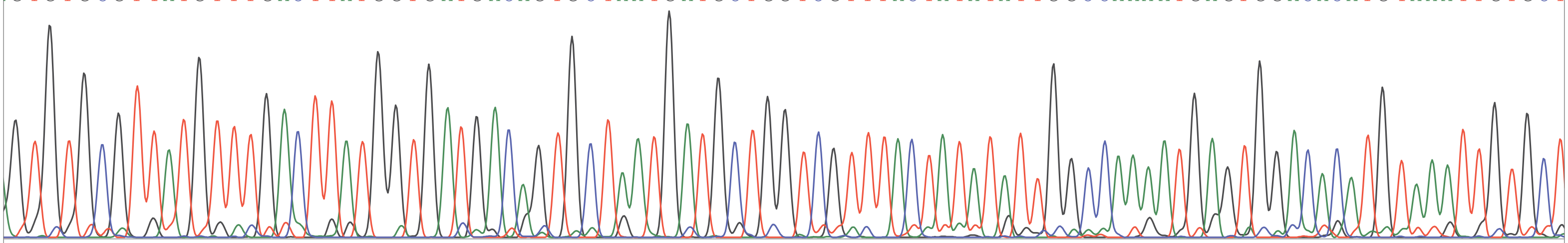

200 210 220 230 240 250 260 270 280 290
TTTGTGGTCAGCGGAAGTGTTTGAAGATGATGACGGATCACTAAAGAATGTGCATATGGCTGTGATCAGGGGCAAGCAGCTGAGATGTGAATTCTGCCAAAA

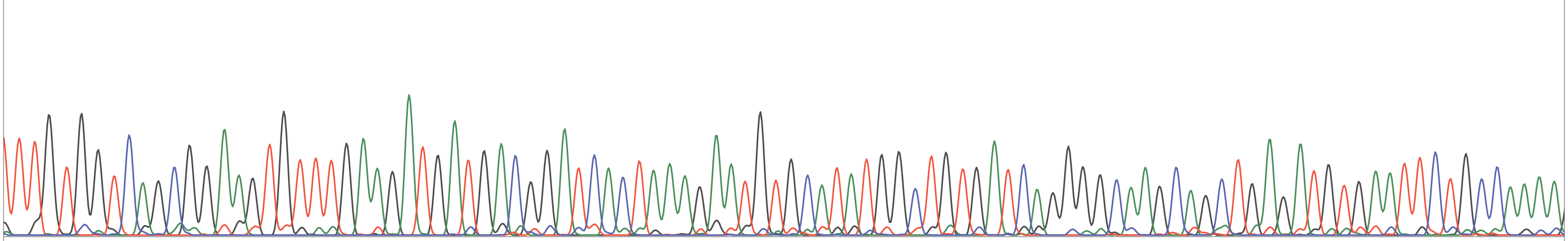

300 310 320 330 340 350 360 370 380 390 400
GCCAGGAGCCACCGTGGGTTGCTGTCTCACATCCTGCACCAGCAACTATCACTTCATGTGTTCCCGAGCCAAGAACTGTGTCTTTCTGGATGATAAAAAAGTAT

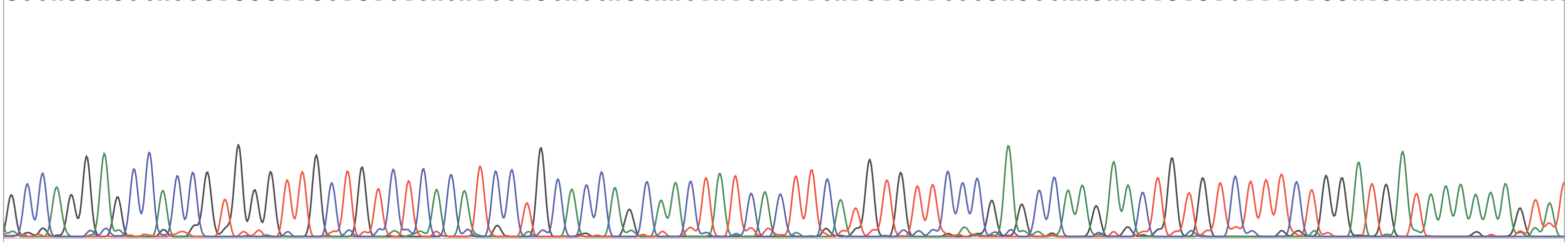

410 420 430 440 450 460 470 480 490 500
TATTGCCAACGACATCGGGATTTGATCAAAGGCGAAAATTCTAAGAGTGCCCAGGGTCTGGCTGGTCTTCGAAACCTTGGGAACACGTGCTTCATGAACTCA

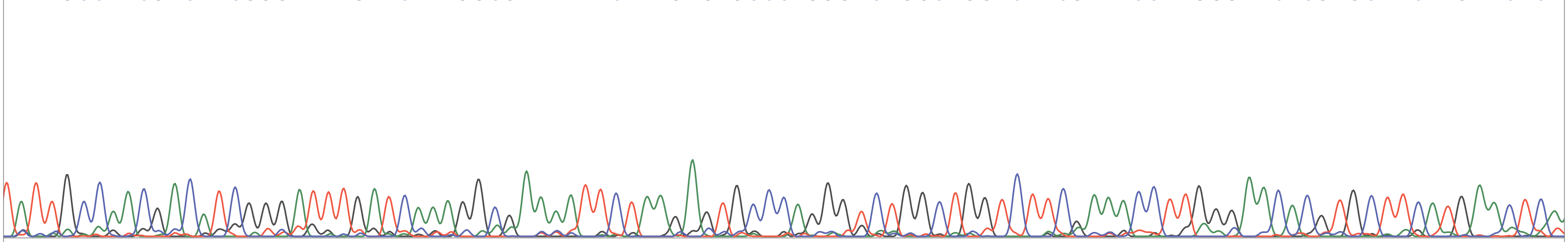

510 520 530 540 550 560 570 580 590 600
ATTCTGCAGTGCCTGAGCAACACTCGGGAGTTGAGAGATTACTGCCTCCAGAGGCTCTACATGCGGGACCTGCACCACGGCAGCAATGCACACACAGCCC

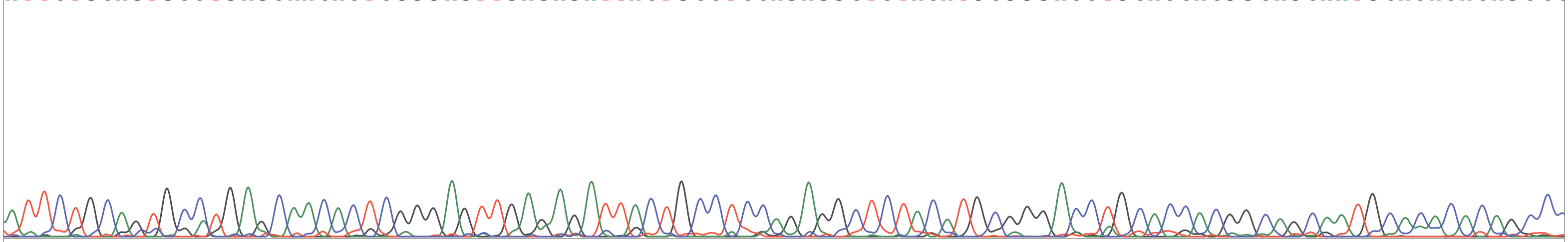

610 620 630 640 650 660 670 680 690 700
CTCGTGGAAGAGTTTGCAAAACTAATTCAGACCATATGGACTTCATCCCCCAATGATGTGGTGAGCCCCATCTAAATTATGTTGTTTTTTGTTGACCTCC

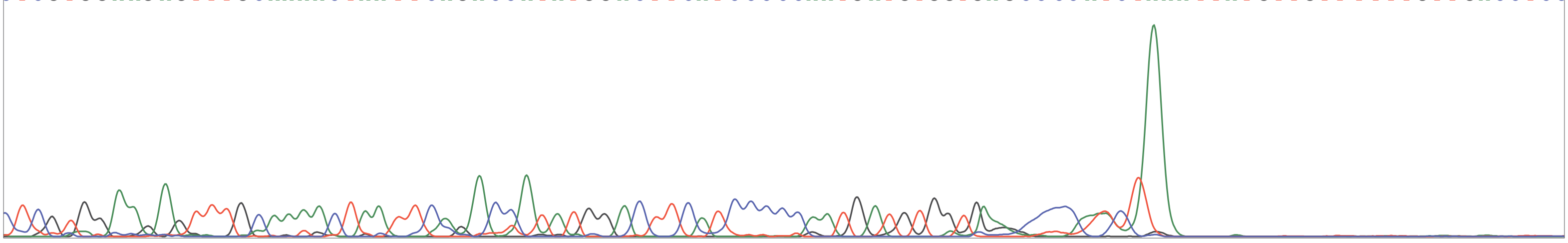

File: MPRIP-JAK2_FOR_93DG07.ab1
Comment: Kundensequenzierung-EasySeq
387 bases in 4715 scans

Run Ended: Jan 16, 2018, 1:54:04
Sequence: 37110222
Page 1 of 1

Signal G:430 A:393 T:635 C:745
Lane: 60 Base spacing 14.71

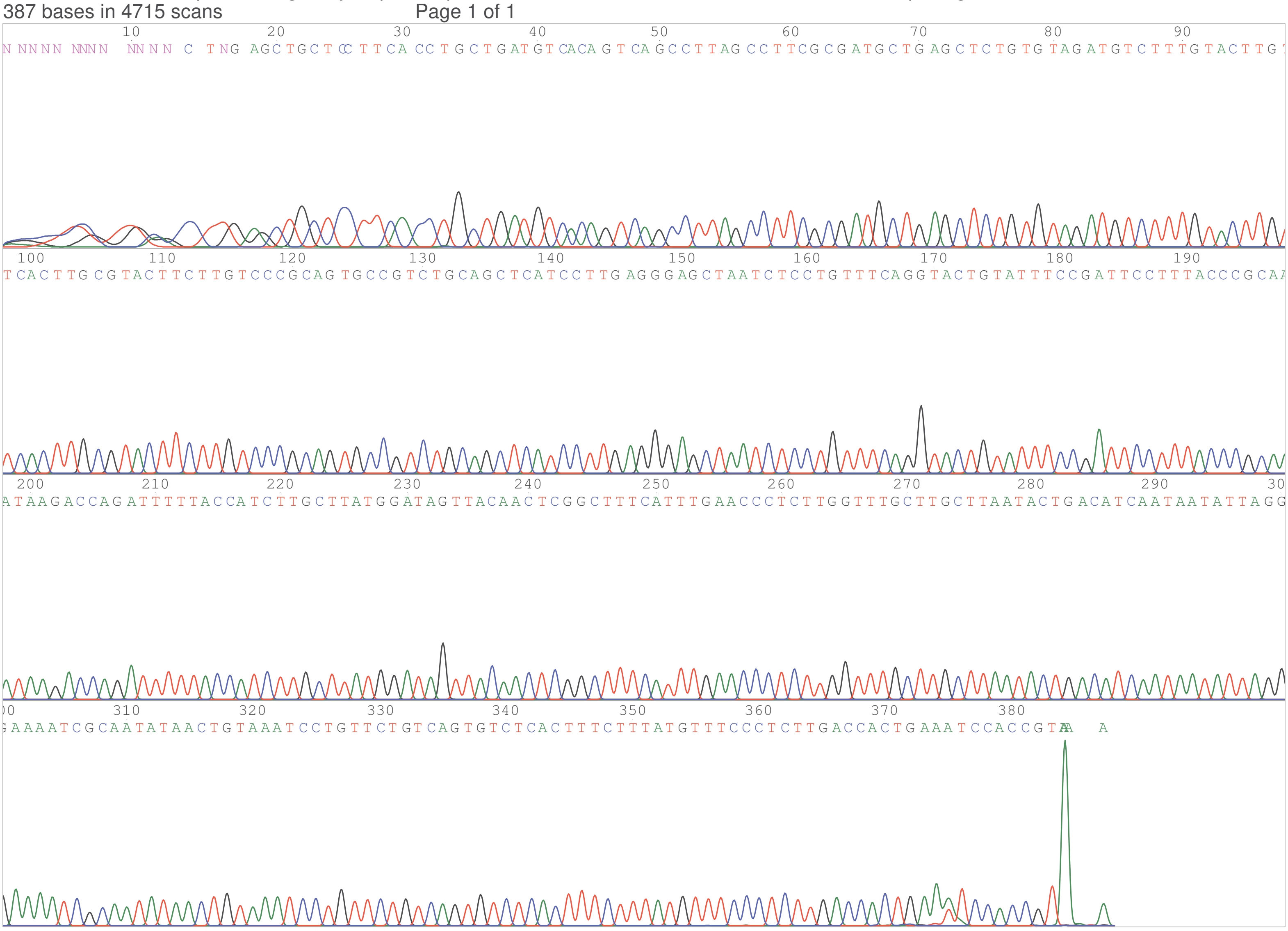

File: NUP214-ABL1_FOR_98EF26.ab1
Comment: Kundensequenzierung-EasySeq
673 bases in 8158 scans

Run Ended: Dec 16, 2017, 3:30:41
Sequence: 36694968

Signal G:2578 A:4479 T:4856 C:6883
Lane: 90 Base spacing 14.46

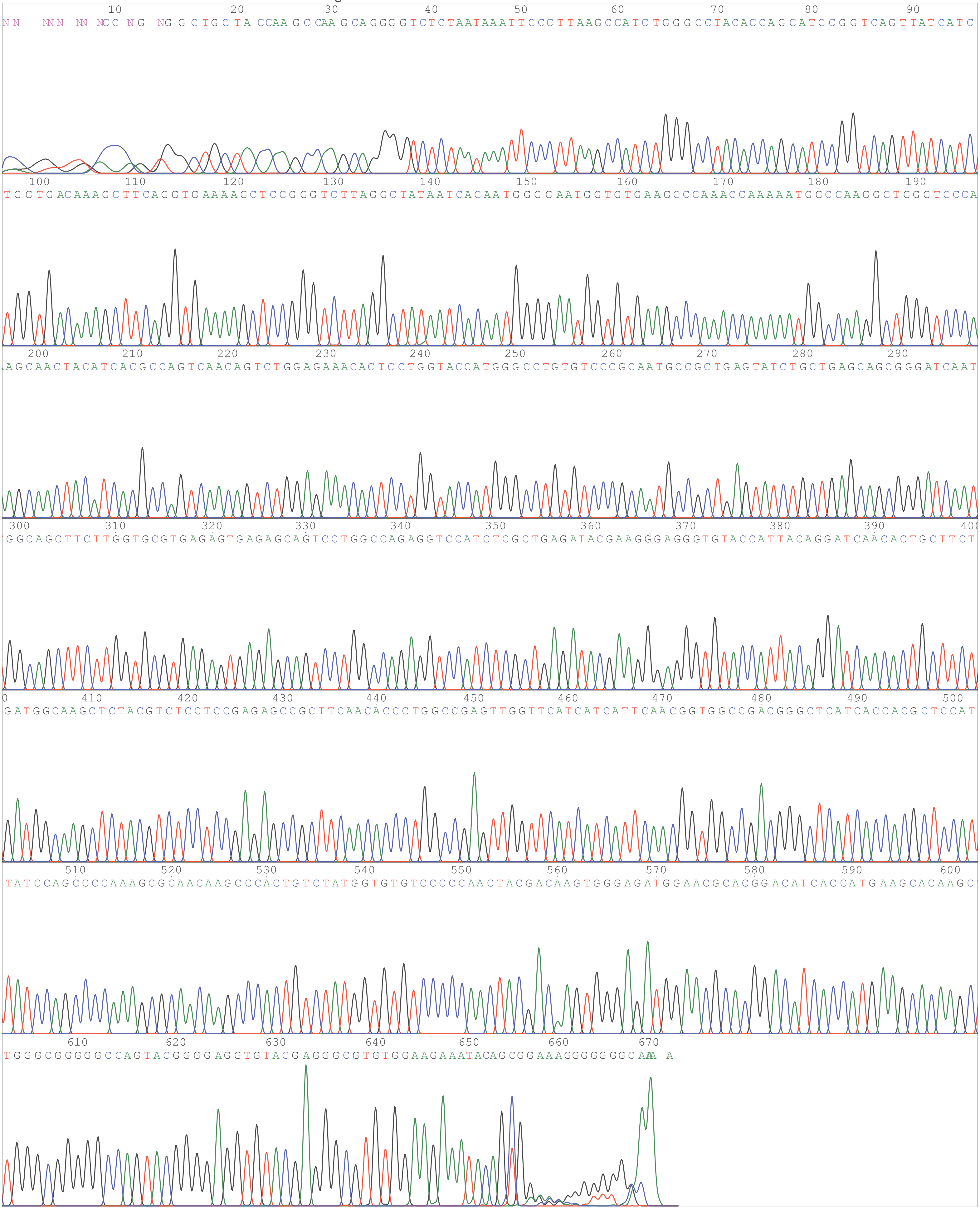

File: PAX5-C20ORF112_FOR_81FJ76.ab1 | Run Ended: Oct 6, 2017, 22:53:16 | Signal G:2832 A:2296 T:2705 C:4103

Comment: Kundensequenzierung-EasySeq | Sequence: 35039596 | Lane: 67 Base spacing 14.51

473 bases in 5792 scans | Page 1 of 1

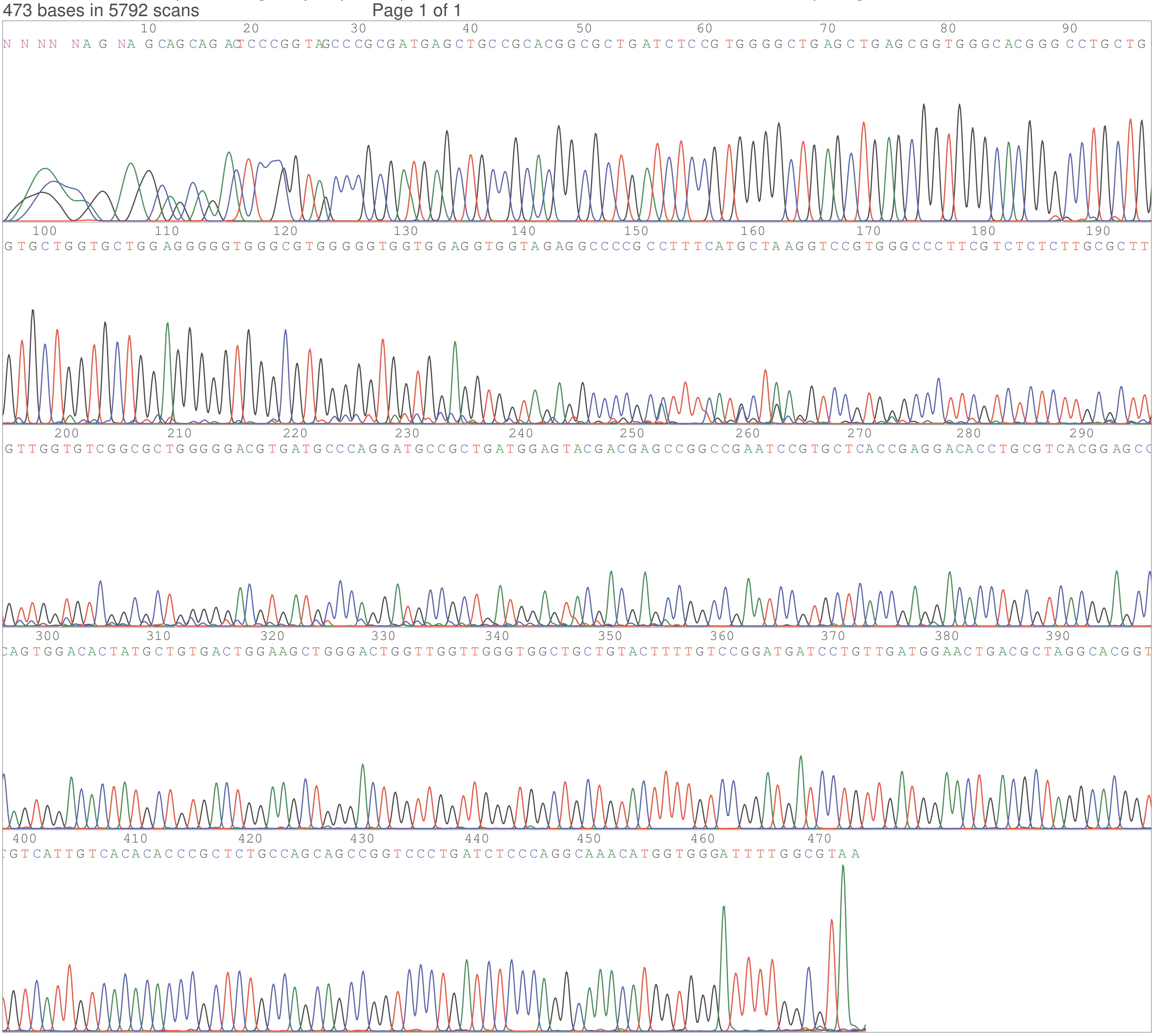

File: PAX5-JAK2_FOR_81FI58-M.ab1
Comment: Kundensequenzierung-EasySeq
308 bases in 3753 scans

Run Ended: Sep 4, 2017, 19:28:19
Sequence: 34331173
Page 1 of 1

Signal G:1564 A:3283 T:4648 C:4424
Lane: 89 Base spacing 15.22

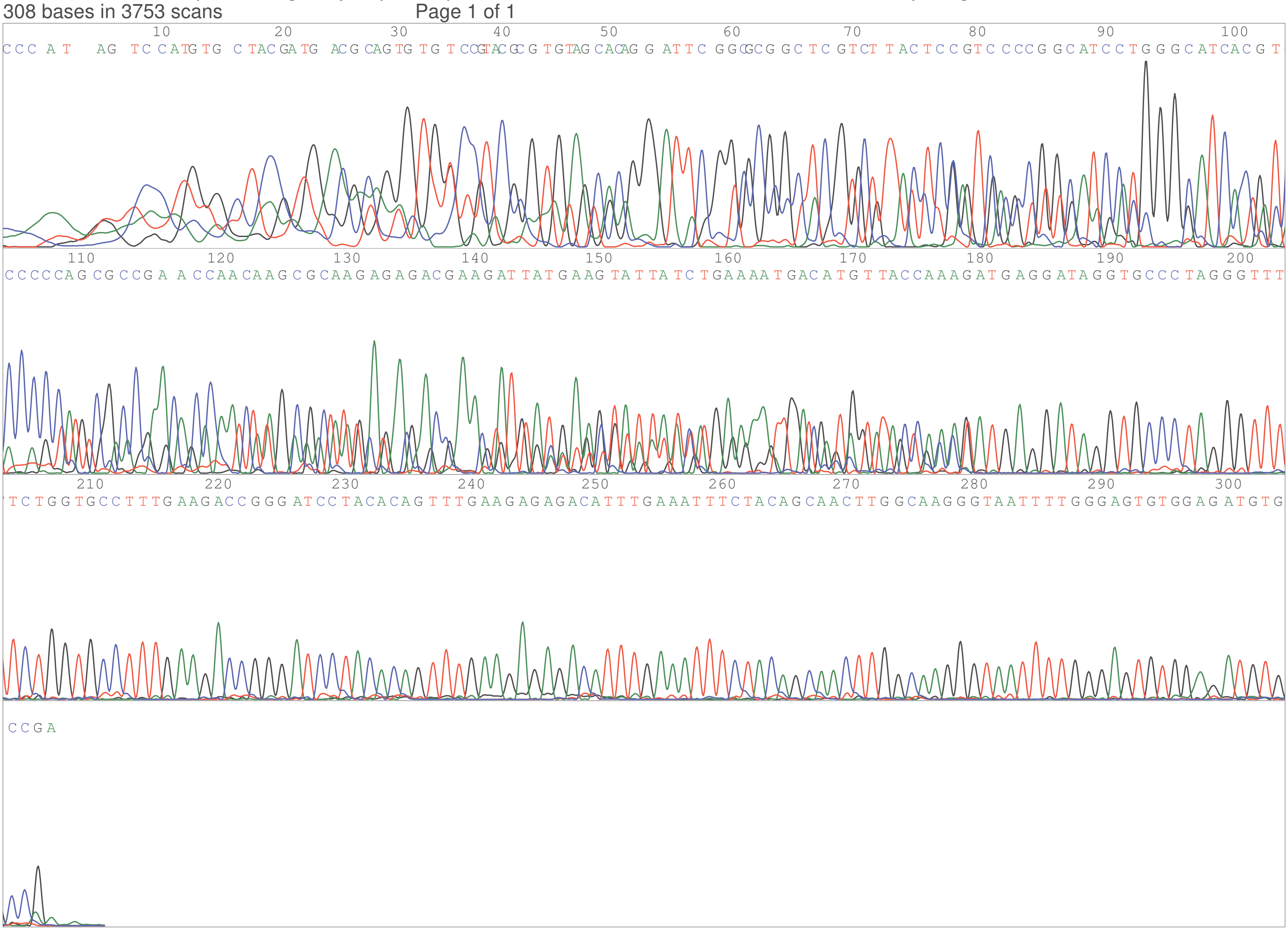

File: PAX5-MBNL1_FOR_81FJ29.ab1
Comment: Kundensequenzierung-EasySeq
756 bases in 9193 scans

Run Ended: Sep 26, 2017, 22:05:00
Sequence: 34808734

Signal G:560 A:1028 T:933 C:1728
Lane: 58 Base spacing 14.23

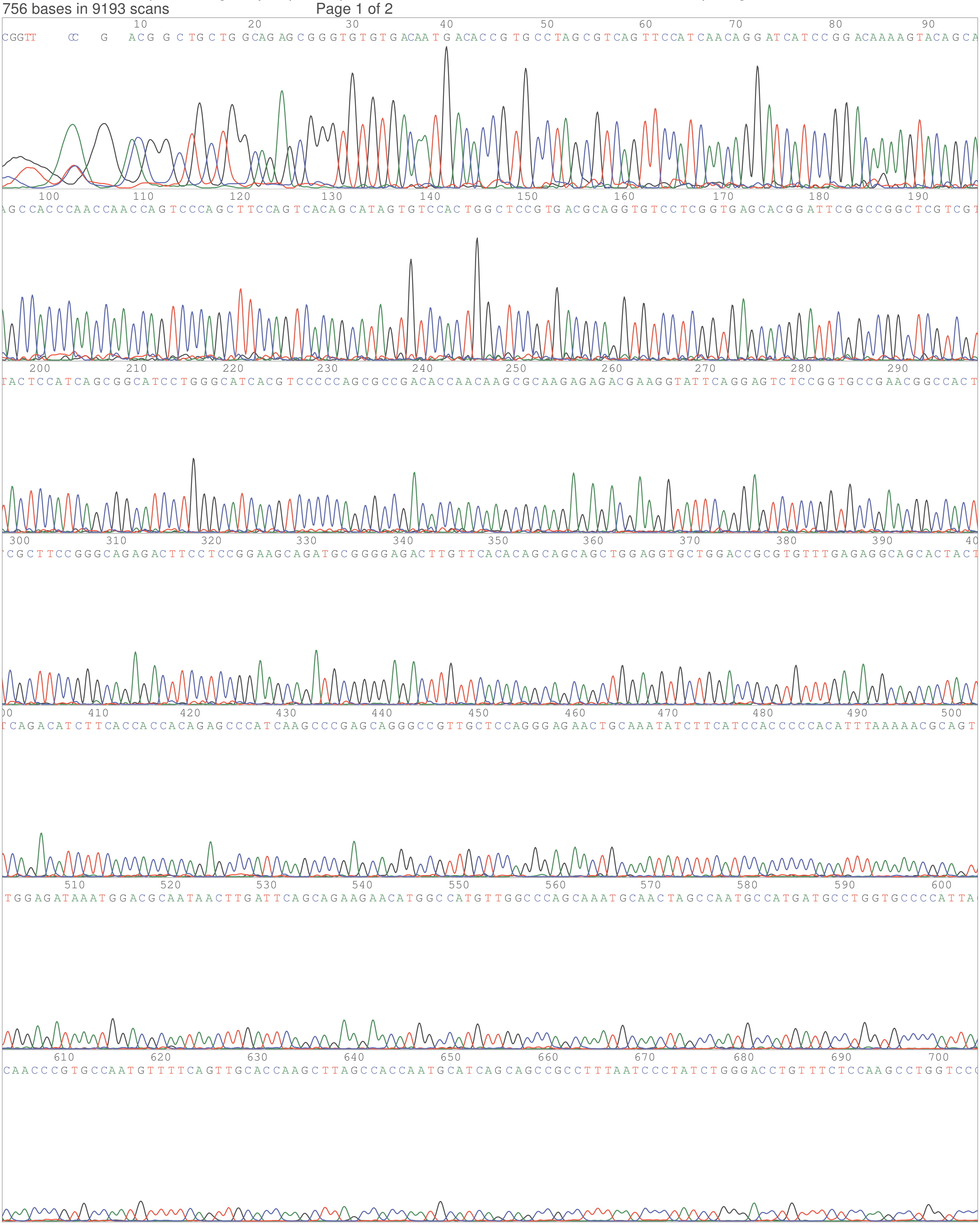

File: RB1-RCBTB2_FOR_98EG11.ab1
Comment: Kundensequenzierung-EasySeq
276 bases in 3499 scans

Run Ended: Apr 24, 2018, 3:57:36
Sequence: 39480497
Page 1 of 1

Signal G:2248 A:3160 T:3509 C:4888
Lane: 23 Base spacing 14.76

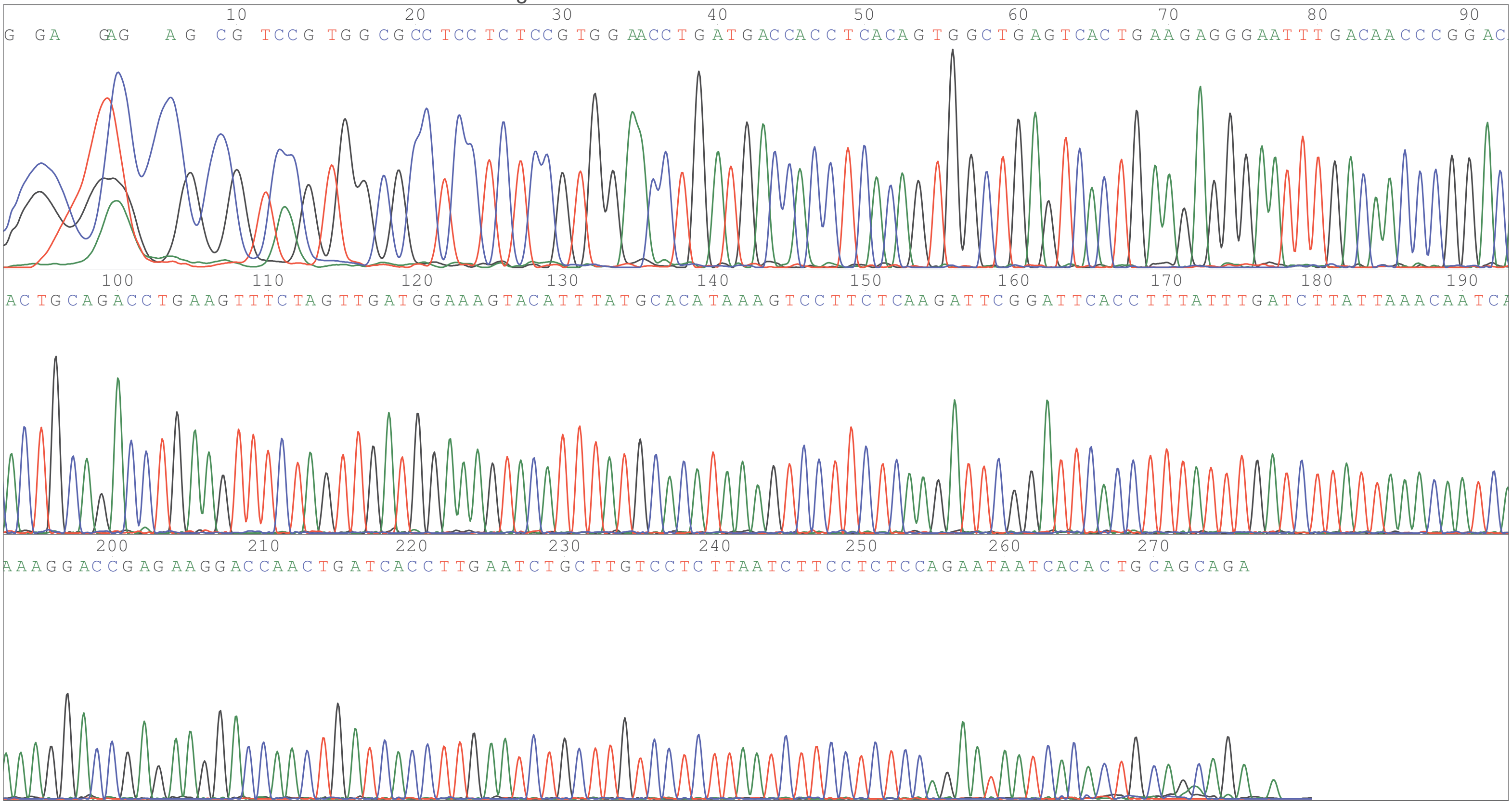

File: TCF3-HLF_FOR_95FF26.ab1
Comment: Kundensequenzierung-EasySeq
332 bases in 4226 scans

Run Ended: Apr 20, 2017, 23:28:17
Sequence: 31286226
Page 1 of 1

Signal G:1357 A:1376 T:1434 C:2788
Lane: 40 Base spacing 16.84

10 20 30 40 50 60 70 80 90 100

GNNNNNNNNNNNNNNGGCACCCTCCCTGACCTGTCTCGGCCTCCCGACTCCTACAGTGCCTCTCCCCTGCCTCCAGCTCCCCTGCTCGCTTTCAGAAGCA

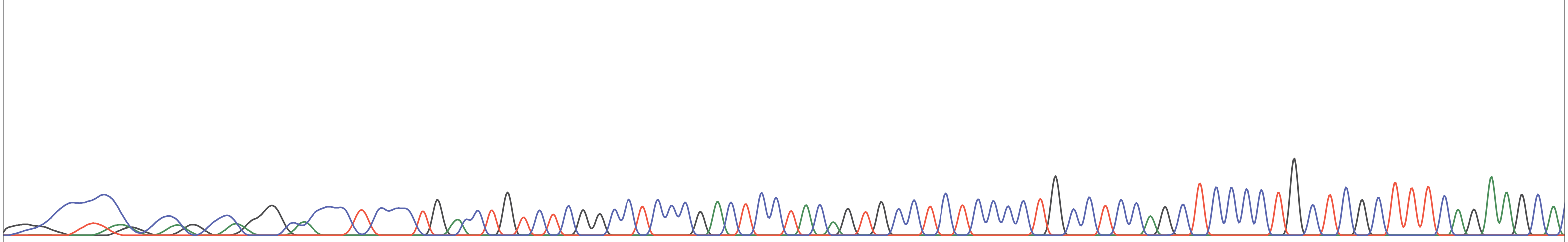

100 110 120 130 140 150 160 170 180 190

CAGACAACAAATTCGGCCAACAACCATTGCACACCTGCCCTAGCTTGCTCACCCAGGTATTCTTCAAAGAGCAGCCTCCTCCCTCCTACCCAGAA

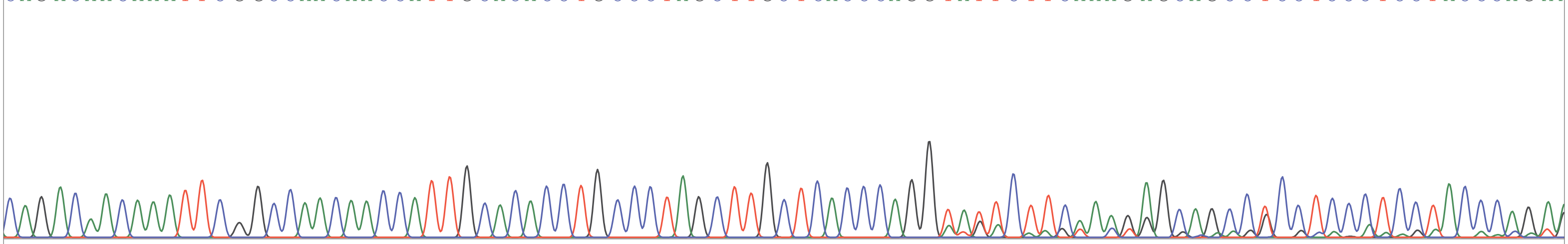

200 210 220 230 240 250 260 270 280

AGAATTCTGGATGACAAGTACTGGGCAAGGCGCAAAAAGAACAACATGGCAGCCAAGCGCTCCCGCGACGCCCGGAGGCTGAAAGAGAAACAGA

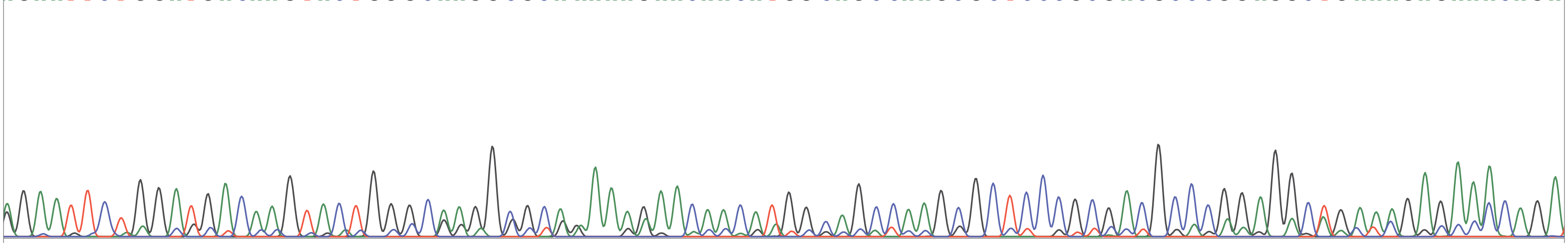

290 300 310 320 330

TCGCCATCCGGGCCTCGTTCCTGGAGAAGGAGAACTCGGCCCAA

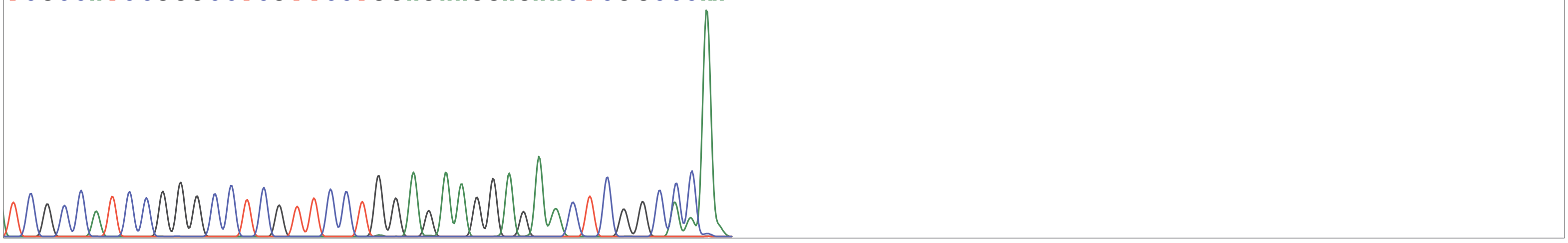

File: TCF3-OAZ1_FOR_81GA68.ab1
Comment: Kundensequenzierung-EasySeq
316 bases in 3824 scans

Run Ended: Nov 16, 2017, 18:42:28
Sequence: 35920286
Page 1 of 1

Signal G:548 A:592 T:400 C:1066
Lane: 38 Base spacing 15.08

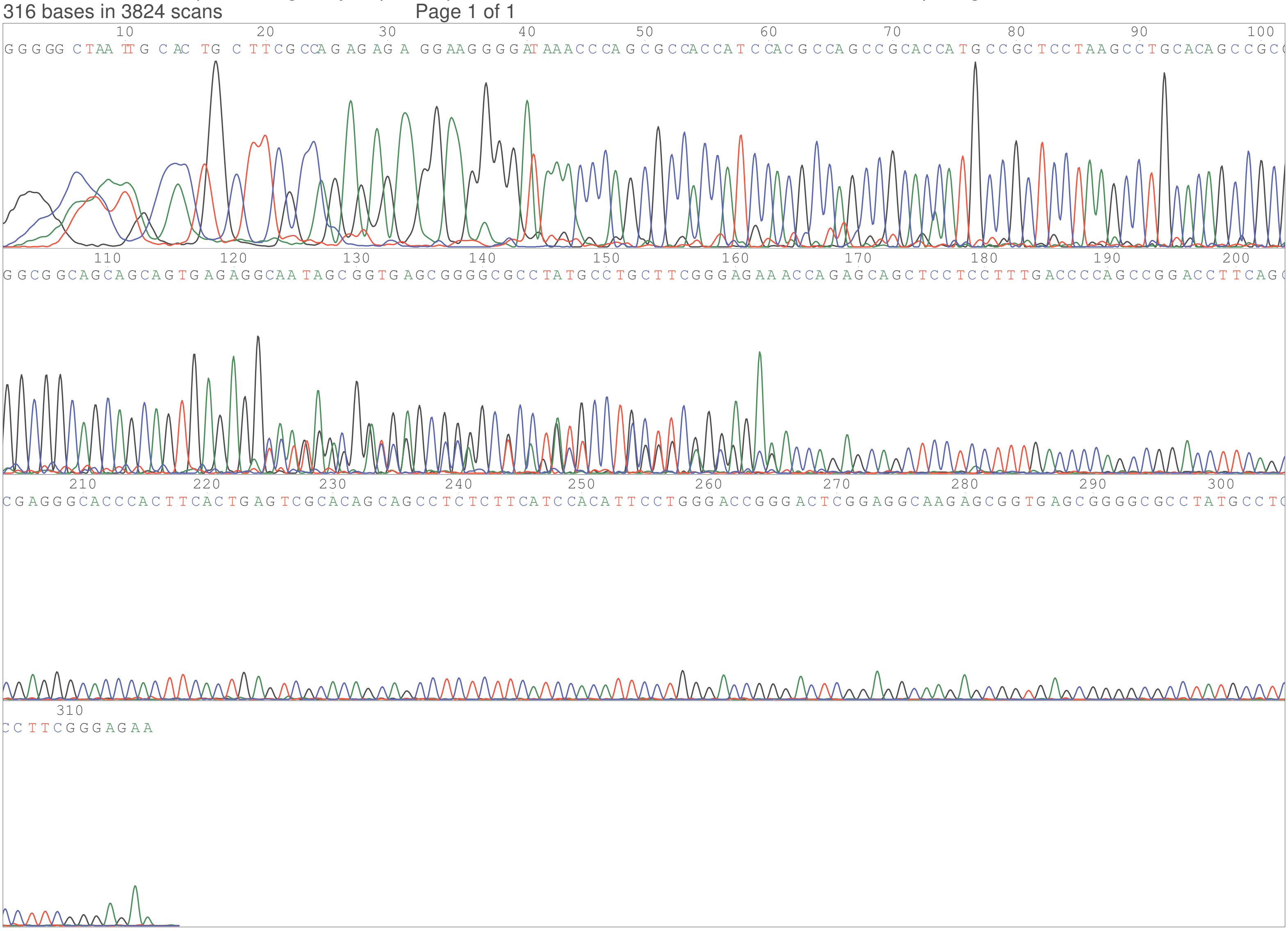

File: ZNF384-EP300_FOR_93DH34.ab1
Comment: Kundensequenzierung-EasySeq
524 bases in 6390 scans

Run Ended: Mar 5, 2018, 7:44:35
Sequence: 38358746
Page 1 of 1

Signal G:251 A:299 T:261 C:475
Lane: 70 Base spacing 15.07

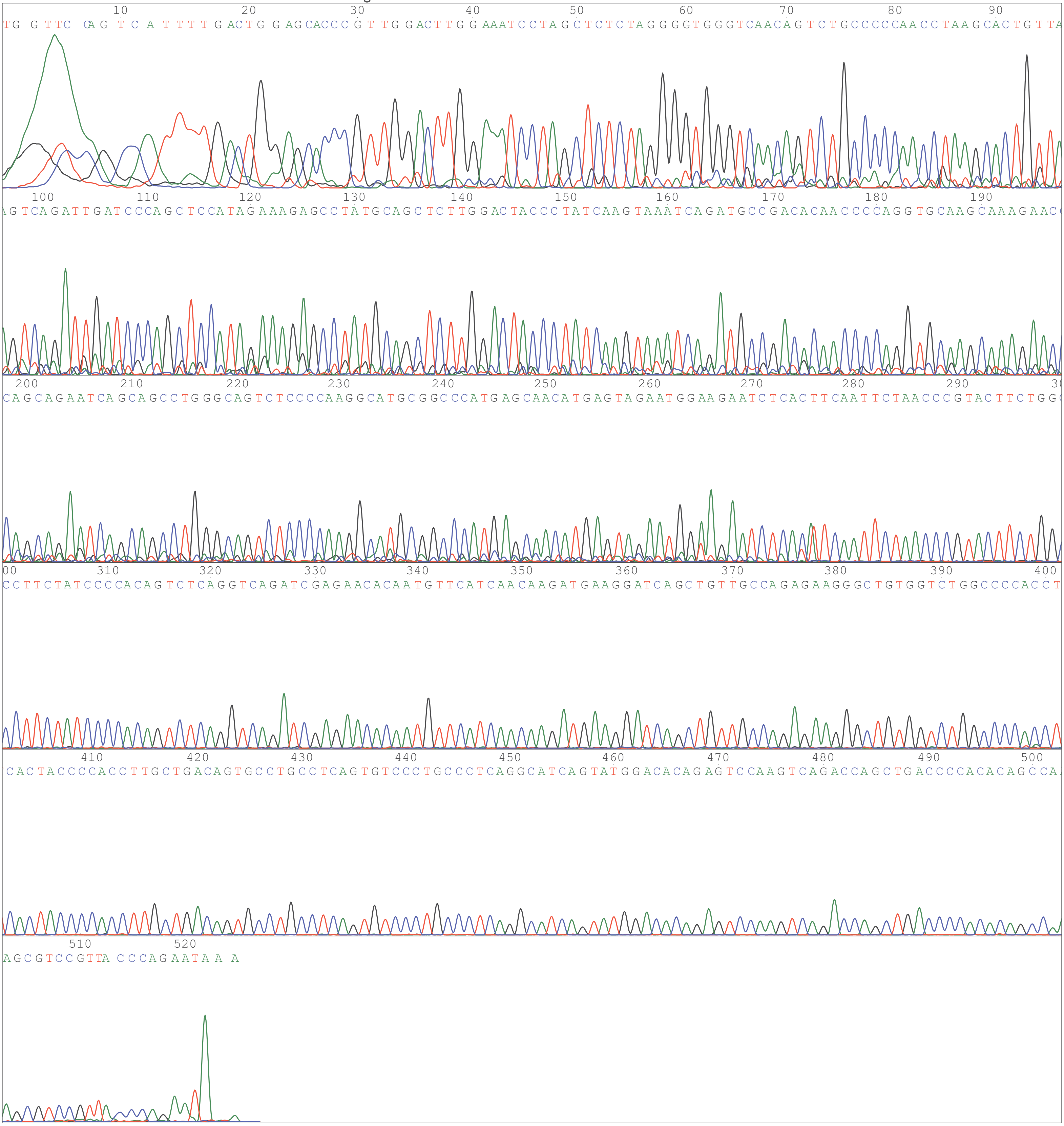

File: ZNF384-TCF3_FOR_81FI62.ab1
Comment: Kundensequenzierung-EasySeq
661 bases in 8068 scans

Run Ended: Sep 4, 2017, 19:28:54
Sequence: 34331308

Signal G:512 A:585 T:581 C:1107
Lane: 45 Base spacing 14.80

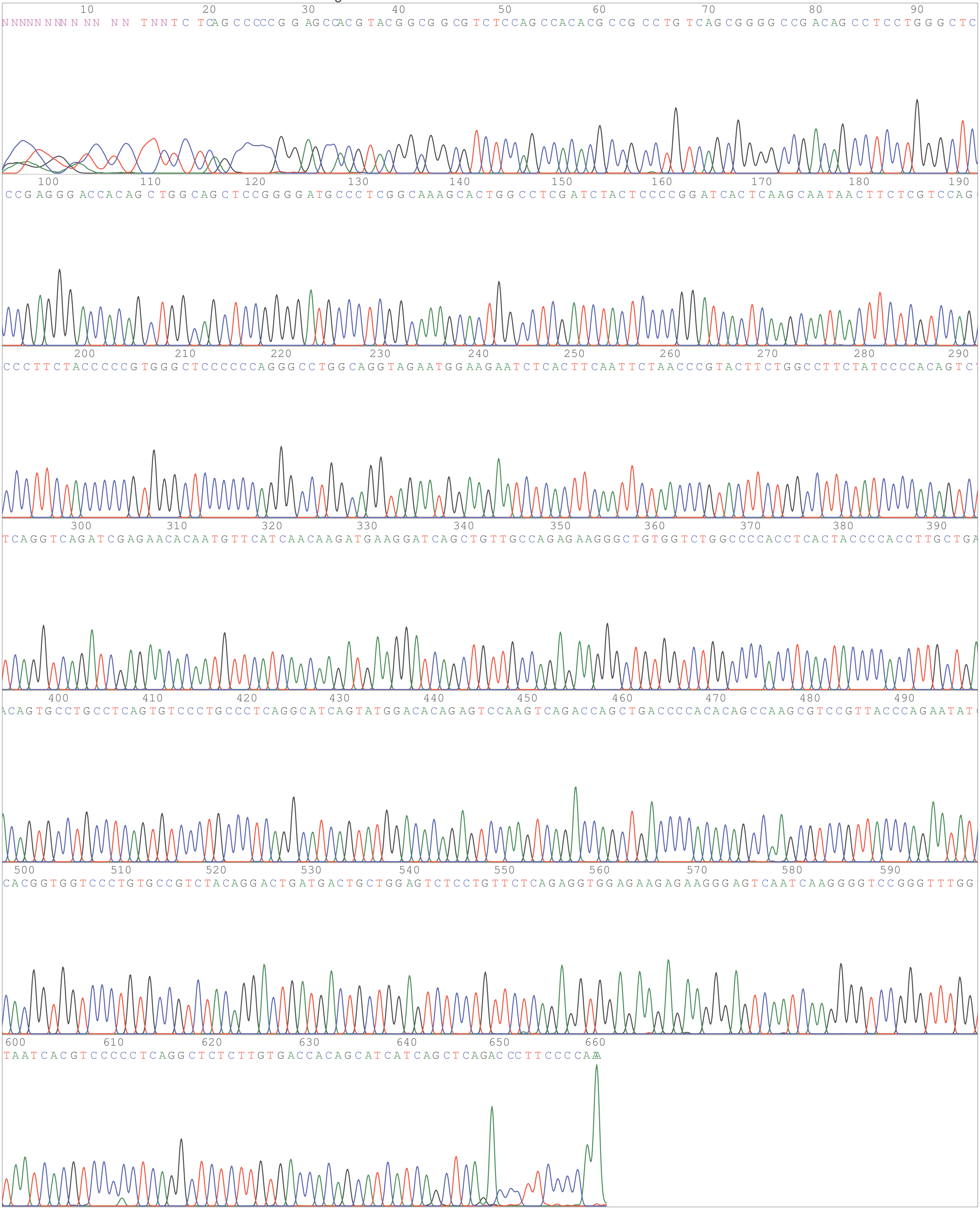

Supplement: Supplemental Digital Content [file hs9-3-e250-s001.pdf]
